# Deletion of genes involved in the ketogluconate metabolism, Entner-Doudoroff pathway, and glucose dehydrogenase increase local and invasive virulence phenotypes in *Streptococcus pneumoniae*

**DOI:** 10.1371/journal.pone.0209688

**Published:** 2019-01-08

**Authors:** Fen Z. Hu, Jarosław E. Król, Chen Hsuan Sherry Tsai, Rory A. Eutsey, Luisa N. Hiller, Bhaswati Sen, Azad Ahmed, Todd Hillman, Farrel J. Buchinsky, Laura Nistico, Bethany Dice, Mark Longwell, Edward Horsey, Garth D. Ehrlich

**Affiliations:** 1 Center for Genomic Sciences, Institute for Molecular Medicine and Infectious Disease, Drexel University College of Medicine, Philadelphia, PA, United States of America; 2 Department of Microbiology and Immunology, Drexel University College of Medicine, Philadelphia, PA, United States of America; 3 Department of Otolaryngology-Head and Neck Surgery, Drexel University College of Medicine, Philadelphia, PA, United States of America; 4 Center for Advanced Microbial Processing, Institute for Molecular Medicine and Infectious Disease, Drexel University College of Medicine, Philadelphia, PA, United States of America; 5 Department of Biological Sciences, Carnegie Mellon University, Pittsburgh, PA, United States of America; 6 Center of Excellence in Biofilm Research, Allegheny Health Network, Pittsburgh, PA, United States of America; Instituto Butantan, BRAZIL

## Abstract

*Streptococcus pneumoniae* displays increased resistance to antibiotic therapy following biofilm formation. A genome-wide search revealed that SP 0320 and SP 0675 (respectively annotated as 5-keto-D-gluconate-5-reductase and glucose dehydrogenase) contain the highest degree of homology to CsgA of *Myxococcus xanthus*, a signaling factor that promotes cell aggregation and biofilm formation. Single and double *SP 0320* and *SP 0675* knockout mutants were created in strain BS72; however, no differences were observed in the biofilm-forming phenotypes of mutants compared to the wild type strain. Using the chinchilla model of otitis media and invasive disease, all three mutants exhibited greatly increased virulence compared to the wild type strain (increased pus formation, tympanic membrane rupture, mortality rates). The *SP 0320* gene is located in an operon with *SP 0317*, *SP 0318* and *SP 0319*, which we bioinformatically annotated as being part of the Entner-Doudoroff pathway. Deletion of *SP 0317* also resulted in increased mortality in chinchillas; however, mutations in *SP 0318* and *SP 0319* did not alter the virulence of bacteria compared to the wild type strain. Complementing the *SP 0317*, *SP 0320 and SP 0675* mutant strains reversed the virulence phenotype. We prepared recombinant SP 0317, SP 0318, SP 0320 and SP 0675 proteins and confirmed their functions. These data reveal that disruption of genes involved in the degradation of ketogluconate, the Entner-Doudoroff pathway, and glucose dehydrogenase significantly increase the virulence of bacteria *in vivo*; two hypothetical models involving virulence triggered by reduced in carbon-flux through the glycolytic pathways are presented.

## Introduction

*Streptococcus pneumoniae* (pneumococcus) colonizes the human nasopharynx [1], and is also an upper respiratory tract pathogen that is often the causative agent of both acute and chronic otitis media with effusion [2–6]. A European Day Care study demonstrated that greater than 95% of children were colonized by *S*. *pneumoniae* during the study [7]. Moreover it has been reported that in developed and developing countries alike, essentially all children become nasopharyngeally colonized during the first year of life [8].

The pneumococcus can also cause life-threatening invasive diseases including pneumonia, bacteremia and meningitis that affect millions of persons annually throughout the world [9–14]. Infections, both acute and chronic, localized and invasive, caused by *S*. *pneumoniae* have become increasingly difficult to treat, due to the acquisition of genetic determinants rendering it antibiotic resistance and its ability to form a biofilm that is metabolically recalcitrant to antibiotic therapy [15–19].

Formation of a biofilm is initiated with the attachment of bacterial cells to the surface followed by cell aggregation and growth [20]. In the Gram-negative rod *Myxococcus xanthus*, cell aggregation and sporulation are induced by the extracellular C-signaling factor (CsgA) [21–23]. Previous study has shown that mutants lacking CsgA are unable to aggregate and sporulate [23], and CsgA has been proposed to induce aggregation via a contact-dependent mechanism [21, 24]. The increase in number of bacterial cells during growth and aggregation allows for the more closely packed cells to promote C-signaling [23].

In this study, we characterized two genes *SP 0320* and *SP 0675* with the highest similarity to CsgA in pneumococcus as well as *SP 0317*, *SP 0318*, *SP 0319* genes cotranscribed with the *SP 0320*, and studied the effect of deleting these genes via examination of growth characteristic, biofilm formation, and virulence *in vivo*.

## Materials and methods

### Bacterial strains and growth conditions

A low passage clinical strain, BS72, of *S*. *pneumoniae* serotype 23, isolated from the nasopharnyx of a child suffering from otitis media [17, 25], was used to make the insertional-deletion mutants in which either an erythromycin or a tetracycline resistance cassette was inserted to replace the gene of interest. Erythromycin at 0.25 μg/ml or 1.0 μg/ml was used for culturing mutant strains (*SP 0320*::*ery* and *SP 0320*:*SP0675* double mutant) in Todd Hewitt broth (THB) or Todd Hewitt agar (THA), respectively. Tetracycline (10 μg/ml) was used to grow the *SP 0317*, *SP 0318*, *SP 0319*, and *SP 0675* mutants. For complemented mutants, the originally disrupted gene was replaced along with a spectinomycin resistance cassette. The complemented strains were cultured in THB plus 75 μg/ml spectinomycin or on THA plus 300 μg/ml spectinomycin. For verifying the presence of α-hemolysis, tryptic soy agar plates with 5% sheep’s blood (BA) were used. For overexpression of recombinant proteins, the Magic Media *E*. *coli* expression system (Invitrogen) plus 50 μg/ml carbenicillin was used. Although primers were designed based on the *S*. *pneumoniae* TIGR 4 or R6 sequences, mutations were introduced into *S*. *pneumoniae* serotype 23 strain. S4 Table (S4 Table) shows the accession numbers of genes in *S*. *pneumoniae* serotype 23 strain that correspond to the orthologous genes in TIGR 4 strain.

### Mutagenesis of *SP 0320*, *SP 0675*, *SP 0317*, *SP 0318*, and *SP 0319* using tripartite ligation

To study the function of *SP 0320*, an insertion-deletion mutant was constructed using a tripartite ligation protocol described previously [13, 14, 26]. Briefly, an erythromycin resistance cassette (Erm^R^) was amplified by PCR using primers, PcErm-ApaI-F and PcErm-BamHI-R (Primers are listed in S5 Table) and Pc-Erm cassette as template (S6 Table, a kind gift from Dr. Morrison, UIC) [27, 28]. This was followed by digestion of Erm^R^ cassette with *ApaI* and *BamHI*. Using primers, SP 0320-Flnk1-F and SP 0320-Flnk1-BamHI-R, a region of DNA downstream of *SP 0320* including a small portion of the 5’ end of *SP 0320* was amplified using *S*. *pneumoniae* Type 23 genomic DNA as template (flank 1). The flank 1 product was digested with *BamHI*. Using primers, SP 0320-Flnk2-ApaI-F and SP 0320-Flnk2-R, a region of DNA upstream of *0320* including a small portion of the 3’ end of *SP 0320* was amplified (flank 2) and this product was digested with *ApaI*. The three products (Erm^R^ cassette, flank1 and flank2) were ligated using T4 DNA ligase, and the tripartite ligation mixture was naturally transformed into *S*. *pneumoniae* serotype 23 wild type strain in the presence of a competent stimulating peptide, CSP1 (a kind gift from Dr. Morrison, UIC) [29–31]. The *SP 0320* insertion-deletion mutant was obtained by allelic exchange. The mutation was confirmed by PCR using primers SP 0320-MutnConfirm-F and SP 0320-MutnConfirm-R and also by sequencing.

Mutagenesis of *SP 0675*, *SP 0317*, *SP 0318*, and *SP 0319* was carried out as described above except that a tetracycline resistance cassette, amplified from PcTet fragment (S6 Table, a kind gift from Dr. Morrison, UIC) using primers PcTet-ApaI-F1 and PcTet-BamHI-R1, was used to create the deletion [27, 28]. All mutations were confirmed by PCR and DNA sequencing.

### Construction of the *SP 0320* and *SP 0675* double mutant

The genomic DNA of the *SP 0675* mutant was isolated using the Bactozol kit (Molecular Research Center Inc, Cincinnati, Ohio). The purified *SP 0675* mutant DNA was transformed into the *SP 0320* knockout strain in the presence of CSP1. Transformants from the *SP 0320*:*0675* double mutant construct experiment were cultured on media containing both erythromycin and tetracycline and were confirmed by PCR amplification and sequencing using primers SP 0320-Mutn-Confirm-F, SP 0320-Mutn-Confirm-R, SP 0675-Mutn-Confirm-F, and SP 0675-Mutn-Confirm-R (S5 Table).

### Complementation of *SP 0317* mutation using a tetrapartite ligation (Fig 1 and S5 Table)

**Fig 1:**
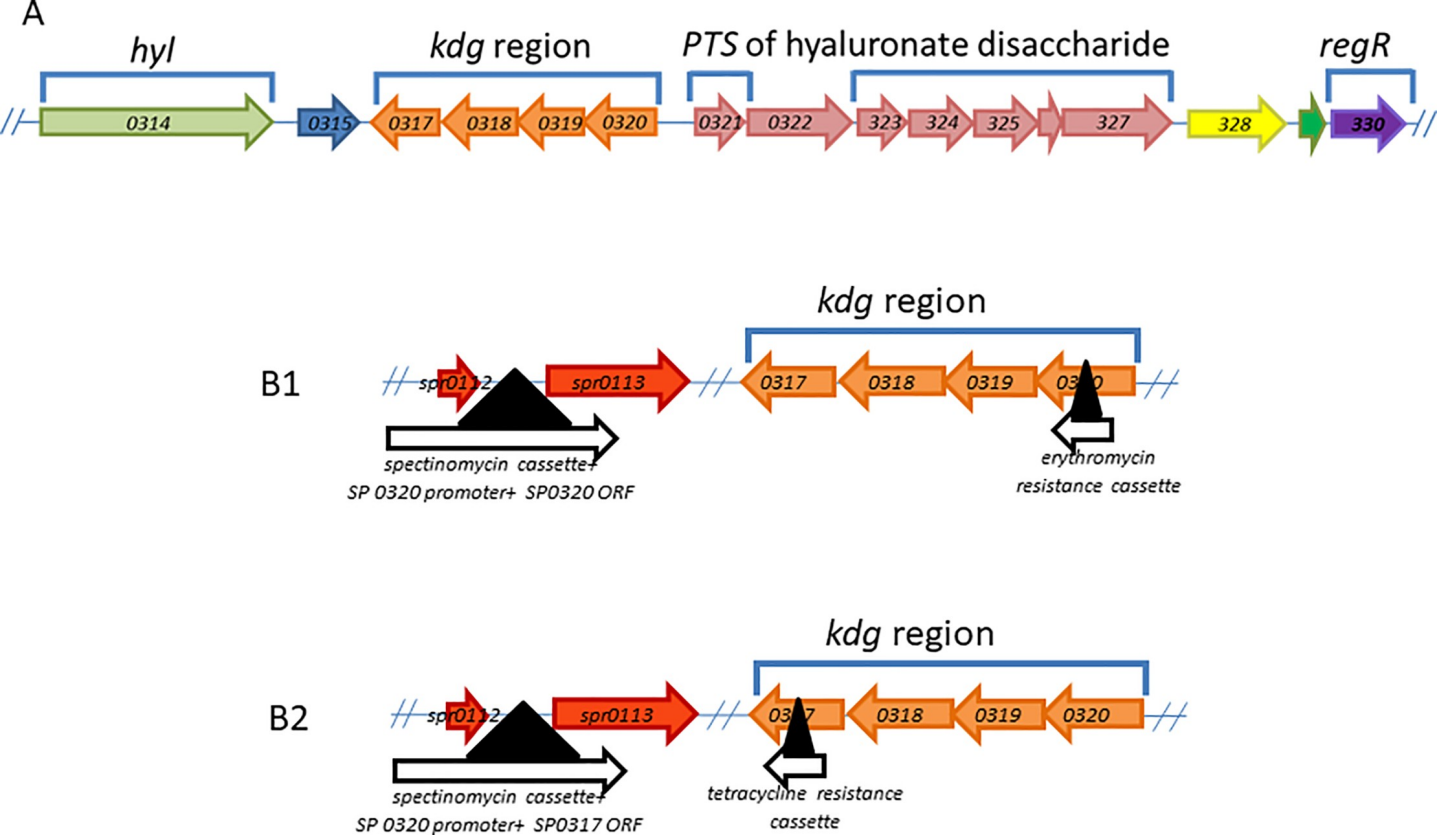
The *kdg* region of *S*. *pneumoniae* TIGR 4 strain. The diagrams are not drawn to scale. Transcriptional directions of genes are shown by the direction of arrows. Loci names correspond to *S*. *pneumoniae* TIGR 4 strain, except for s*pr 0112* and *spr 0113* (not found in strain TIGR4), which are the designated names for strain R6, and their corresponding names in strain serotype 23 are *ZP_01834243*.*1* and *ZP_01834246*.*1*. Genes that constitute the *kdg* region are colored in orange (*SP 0317–0320*). Panel A: A pseudogene (colored in blue) is directly downstream to the *kdg* region. Further downstream to the *kdg* region is *SP 0314*, a gene that encodes the hyaluronate lyase (*hyl*) (colored in green). Upstream to the *kdg* region (colored in pink) are genes that encode a PTS transport system (*SP 0321*, *SP 0323*, *SP 0324*, *SP 0325*) and glucuronyl hydrolase (*SP 0322*). The functions of the PTS transport system and glucuronyl hydrolase are to respectively transport hyaluronate disaccharides into the cell and to further break down hyaluronate disaccharide into monosaccharide. Colored in yellow (*SP 0328*), grey (*SP 0329*) and purple (*SP 0330*) respectively represent genes that encode a transpose, protein of unknown function, and RegR (a global regulator). Panel B: Construction of the complemented *SP 0320* (panel B1) and *SP 0317* (panel B2) mutant strains (see Materials and Methods).

To complement the *SP 0317* mutation, the *SP 0317* gene of *S*. *pneumoniae* serotype 23 was amplified using primers SP 0317-F and SP 0317-R (S2 Table) and cloned into pGEM-T easy vector (Promega) to give the construct pGEMT: 0317. Since *SP 0317* is clustered along with *SP 0320*, and does not have its own promoter, the promoter upstream of *SP 0320* was amplified from *S*. *pneumoniae* serotype 23 genomic DNA using primers Prom-SphI-F and Prom-NcoI-R. The promoter fragment was then purified and digested with *SphI* and *NcoI*, and cloned upstream of *SP 0317* between the *SphI* and *NcoI* sites of pGEMT: 0317 construct giving pGEMT: Prom: 0317 construct. The ‘Prom: 0317’ fragment from pGEMT: Prom: 0317 construct was then excised using *SphI* and *NdeI* and used for the four-partite ligation (see below).

Because our aim was to avoid insertional inactivation of any other genes, the tetrapartite ligation product was designed to be inserted into an 805 base pair intergenic region located between two hypothetical genes (*Spr0112* and *Spr 0113*). The *Spr 0112* upstream product was amplified from *S*. *pneumoniae* serotype 23 genomic DNA using Spr 0112 flnk1-F and Spr 0112 flnk1-NcoI-R primers, and the *Spr 0113* downstream product was amplified using Spr 0113 flnk 2-NdeI-F and Spr 0113 flnk2-R1 primers. A spectinomycin resistance cassette (Spec^R^) was amplified with PcSpec-NcoI-F and PcSpec-SphI-R primers using pR412 plasmid as template (S6 Table, a gift from Dr. Morrison, UIC) [28]. After restriction digestion of these four products (Spr 0112 flnk1, Spr 0113 flnk2, Spec^R^ cassette and the ‘Prom-0317’ fragment), a four-partite ligation was carried out. This ligation mixture was transformed into the *SP 0317* knockout mutant.

As a result of allelic exchange, the ‘Prom-0317’ fragment along with the Spec^R^ cassette was introduced into the intergenic region between *Spr 0112* and *Spr 0113* giving rise to the complemented *SP 0317* mutant with a mutated and an intact copy of *SP 0317*. The presence of an intact copy of *SP 0317* was confirmed using SP 0317-Comple-Confirm-F and SP 0317-Comple-Confirm-R. The presence of the mutated copy of *SP 0317* was confirmed using SP 0317-Mutn-Confirm-F and SP 0317-Mutn-Confirm-R (S5 Table).

### Complementation of *SP 0320* (Fig 1) and *SP 0675* mutations using four-partite ligation (S2 Table)

Complementation of *SP 0320* and *SP 0675* knockout strains along with their upstream promoters was performed using DNA amplified from the *S*. *pneumoniae* serotype 23 wild type strain in a similar manner as described for the *SP 0317* complemented mutant. The primers are listed in S6 Table.

### Growth characteristics of the wild type and mutant strains

We determined whether inactivating any of the genes would result in a difference in growth rate compared to the wild type strain. Three to four hour cultures (TSB + 0.5% Yeast Extract) of the wild type and mutant strains were used as inocula to create the starting cultures. The optical density of starting cultures were from 0.01 to 0.05 at 600 nm in THB. Growth of bacteria at 37°C was monitored every 30 minutes at an absorbance of 600 nm using the Tecan GENius Plus plate reader (Tecan Instruments, Salzburg, Austria). Results shown are the average of three independent experiments. Growth rates and generation times were calculated using GrowthRates v2.0 software [32].

### Polysaccharide test

The anthrone reaction was used to quantify the amount of polysaccharide in a tested solution [33]. Anthrone Reagent (Fisher Scientific, USA) was added (0.2 grams) to 100 ml concentrated sulfuric acid. Known concentrations of glucose (0, 25, 50, 100, and 200 mg/L) in water was used as a standard. The culture was grown to OD600nm = 0.15 and diluted 1:1 with THB; grew for 4 hr. Cells were harvested (750ul), washed twice with 1ml of PBS and resuspended in 150 **μ**l of PBS. Cell concentrations were measured at OD 600 nm. Fifty microliter of the solution (test sample, standard, or PBS for a blank) was transferred into centrifuge tubes. Five hundred microliters of anthrone reagent was added into each tube and mixed well. Tubes were boiled at 100°C water bath for 10 min and were cooled for ~10 minutes at room temperature. After that, 100 microliters was used to measure OD 630 nm. The absorbance was normalized with the corresponding culture density. By comparing the ODs of an unknown to those of a standard, the polysaccharide concentration was determined. The results were analyzed by Student T test for statistical significance.

### RNA isolation and quantitative PCR

Single colony was inoculated into 1 ml of THB liquid medium and incubated at 37ºC for 6 h in static 5% CO_2_ incubator. Two microliters culture was used to inoculate 50 ml THB broth. Total RNA was isolated from 12h cultures of all of the mutants and WT strains using RNeasy Mini Kit (Qiagen,). RNA was treated with RNase-Free DNase (Qiagen cat. No. 79254) and tested for the negative PCR results with GyrB primers and the DreamTaq Green PCR Master Mix (ThermoScientific cat. No. K1081). Two micrograms of RNA from mutants and the WT strains were used for reverse transcription (RT) reaction using Superscript III Reverse Transcriptase (Invitrogen). Single reactions contained GyrB-F/GyrB-R and RegR-F/RegR-R or Hyl-F/Hyl-R primer sets (Table A in S5 Table). Fivefold dilutions of the RT reactions were used as a template for a Digital Droplet PCR reaction using a ddPCR SuperMix for probes (Bio-Rad, Cat. No. 186–3025). Droplets were prepared using an Automated Droplet Generator, PCR reactions (95ºC-10 min.; 94ºC-30sec, 50ºC-1min., x39; 95ºC-10min.) were run in C1000 Thermal Cycler (Bio-Rad). DDPCR data were analyzed with the QX200 Droplet Reader and QuantaSoft v.1.6.6 software (Bio-Rad). Three biological replicates were analyzed.

### Biofilm studies using a flow cell system

*S*. *pneumoniae* wild type and mutant strains were injected individually into 1mm x 1mm square glass capillary flow cells (Bio Surface Technologies Corporation, Bozeman, MT). 0.2X THB was pumped into each flow cell at a rate of 0.15 ml/min for 10 days. The flow cells were positioned on a polycarbonate holder and were placed on a stage of a Leica confocal upright microscope so that the biofilms could be imaged *in situ* without interrupting the flow.

### Inoculation of the middle ears of chinchillas with *S*. *pneumoniae* serotype 23 wild type and mutant strains

Using the previously established chinchilla model of otitis media and invasive disease (OMID) [34–36], the virulence of each of the mutant strains was compared to the wild type strain through standardized parameters: an objective clinical scoring system that examines the severity of both middle ear and systemic infection (S3 Table) [37].

Young adult research-grade chinchillas (R and R Chinchilla Ranch, Jenera, Ohio) weighing approximately 500 g were anaesthetized by intramuscular injection of a 0.25 ml cocktail mixture of ketamine (0.25 mg/ml), xylaject (5 mg/ml) and acepromazine (5 mg/ml). After the animals were anaesthetized, a 0.1 ml inoculum containing either 10^3^ CFU/ml of a viable low passage (n < 3) *S*. *pneumoniae* serotype 23 wild type or mutant strain was injected bilaterally via a transbullar approach. Another group of animals were sham inoculated with sterile phosphate buffered saline (PBS). The animals were housed three per cage. The protocols for animal handling were in accordance with the IUCAC guidelines: Protocol #20507; Project #1045108; entitled “Evaluation of the Role of Hypothetical Virulence Genes in *Streptococcus pneumoniae* and *Haemophilus influenzae* infections using the Chinchilla Model of Otitis Media associated with this grant application has been received from the Institutional Animal Care and Use Committee (Assurance # A3222-01): Current Protocol #20507; Project #1045108; entitled “Evaluation of the Role of Hypothetical Virulence Genes in Streptococcus pneumoniae and Haemophilus influenzae Infections Using the Chinchilla Model of Otitis Media” has been approved by the Drexel University Bio-Safety Committee; Action #63246; 09/20/2016). Animals had 24 hour access to food and water; 14hr/10hr light/dark cycling. The degree of otoscopic disease was monitored from day one through to day ten (or until moribundity and euthanasia) for each ear and for each animal, and was scored by a board-certified otolaryngologist using the following established criteria [37, 38]: 0-no change, 1-mild inflammation, 2-moderate inflammation, 3-frank purulence, 4-tympanic membrane rupture. Animals with a moribundity score of 4 (S7 Table) were euthanized. After completion of the ten day otoscopic readings, the animals were heavily sedated with 0.5 ml of the above anaesthetic cocktail, and then euthanized with an intracardiac injection of 0.3 ml potassium chloride. The left and right tympanic bullae were harvested by creating a vertical midline incision down to the bone to expose the dorsal surface of the skull. A piece of the bulla was placed in sterile PBS for DNA extraction and PCR. The lung and the brain were also harvested and stored in sterile PBS. The ear effusions and the lung and brain tissues were homogenized and then aliquoted for PCR and plating. PCR using strain-specific primers was performed to determine persistence in the middle-ear and to look for invasion into the brain and lung. Aliquots for plating were spread on BA plates to test for bacterial recovery of the inoculated strain and to check for the presence/absence of α hemolysis. Statistical analyses of the otoscopic scores were performed using the Kruskal-Wallis rank sum test, and the Fisher’s exact test was used (due to small sample sizes) to analyze the difference in the presence or absence of pus and the survival versus mortality rates for all animals.

Visualization of tissue specimen through FISH was conducted using 16S rRNA gene specific probe labeled with pneumococcal specific Cy3 and FAM. Pneumococcal specific tissue sample yielded the green color, while eubacterial generic specimen showed up in red, and yellow indicated the overly of the two dyes.

### Recombinant expression and purification of the SP 0317, SP 0318, SP 0319, SP 0320 and SP 0675 proteins (S2 Fig)

The *SP 0317*, *SP 0318*, *SP 0319*, *SP 0320* and *SP 0675* genes along with their start and stop codons were PCR amplified and individually cloned downstream of a 6X His tag between the *PmlI* and *AvrII* sites of the pET302/NT-His vector (Invitrogen; Carlsbad, CA). The sequences of primers used for the amplification are shown in S5 Table. The inserts were confirmed by DNA sequencing and plasmids were transformed into *E*. *coli* BL21 DE3 cells (Invitrogen). Each of the N-terminal His tagged proteins was overexpressed by growing the BL21 DE3 cells in the Magic Media *E*. *coli* expression system. The cells were harvested after overnight growth at 37°C and lysed by sonication (four pulses of 10 seconds each at 40% amplitude). After a brief centrifugation at 3000x g for 15 minutes, the supernatant (cytosolic extract) and the cell debris (membrane extract) were independently mixed with loading dye and loaded onto a 12% polyacrylamide gel to check for protein expression. The SP 0319 and SP 0320 proteins were present in the cytosolic extracts, and the SP 0317 and SP 0318 proteins were present both in the cytosolic and the membrane extracts, with the majority in the membrane fraction. SP 0675 was present in the membrane extract. The SP 0317, SP 0318, SP 0319 and SP 0320 proteins were purified using the native purification protocol with the ProBond nickel chelating resin purification kit (Invitrogen). SP 0675 was purified using the hybrid purification protocol. The purity of these proteins was approximately 97%. The protein identities were confirmed by MALDI-TOF (Applied Biosystems Voyager DE-PRO) and the data was searched against *S*. *pneumoniae* TIGR 4 database with Protein Prospector at the PNAS facility at the Medical College of Wisconsin, WI.

### Biochemical assay of SP 0317 (2-keto-3-deoxy-6-phospho-D-gluconate aldolase, EC 4.1.2.14) (S1 Fig)

The sequence homology of SP 0317 indicated that it might function as a 2-keto-3-deoxy-6-phospho-D-gluconate aldolase, and an assay was performed to confirm this. This enzyme catalyzes the conversion of 2-keto-3-deoxygluconate 6-phosphate **(**KDGP) to pyruvate and glyceraldehyde 3-phosphate (G3P). The biochemical assay was designed to check the rate of pyruvate formation [39]. The reaction involves two steps: conversion of KDGP to pyruvate plus G3P by SP 0317 protein, followed by the reduction of pyruvate in the presence of lactic dehydrogenase to give lactate. The reduction of pyruvate is coupled to the oxidation of NADH. NAD^+^ formation from NADH can be monitored spectrophotometrically as a decrease in the A_340_ readings. The reaction mixture consisted of 20 mM KH_2_PO_4_, 0.42 mM NADH (Sigma-Aldrich Corporation, St. Louis, Missouri), 10 mM KDGP (a gift from Dr. Eric J. Toone, Duke University), and 0.1 mg/ml of purified His tagged SP 0317 protein. After a 5 minute incubation at 37 ^o^C, 10 units of commercially available lactic dehydrogenase from *Lactobacillus leichmanii* (Sigma) was added and the incubation continued. The A_340_ readings were recorded every 30 seconds for 15 min. using a Tecan GENius Plus plate reader.

### Biochemical assay of SP 0318 (2-keto-3-deoxygluconokinase, EC 2.7.1.45) (S1 Fig)

Sequence homology searches of SP 0318 indicated that it likely functions as a 2-keto-3-deoxygluconokinase; therefore we set up an assay to test this hypothesis. 2-Keto-3-deoxygluconokinases catalyze the phosphorylation of 2-keto-3-deoxygluconate (KDG) in the presence of ATP to give KDGP. An indirect assay was used to test the phosphorylation activity with KDG as the substrate (a gift from Dr. Sun Bok Lee, Pohang University of Science and Technology) [40]. The reaction mixture consisted of 50 mM Tris-Cl, pH 8.5, 10 mM KDG, 10 mM D-glucose, 1 mM NADP^+^, 10 mM MgCl_2_, 0.1 U/μl of glucose-6-phosphate dehydrogenase, 0.0077 μg/μl of ADP dependent glucokinase, 2.5 mM ATP and 0.16 mg/ml of purified His tagged SP 0318 protein. The increase in the A_340_ reading due to the formation of NADPH was recorded every minute for 30 minutes at 37 ^o^C using the Tecan GENius Plus plate reader. D-Glucose, NADP^+^, ATP and glucose-6-phosphate dehydrogenase were purchased from Sigma. ADP glucokinase was purchased from ABNOVA Corporation, Taiwan.

### Biochemical assay of SP 0320 (5-keto-D-gluconate-5-reductase, EC 1.1.1.69) (S1 Fig)

Sequence homology searches indicated the probable function of SP 0320 as 5-keto-D-gluconate-5-reductase. This enzyme catalyzes the reduction of 5-keto-D-gluconate to D-gluconate with the subsequent oxidation of NADH. The NAD^+^ formation can be monitored by a decrease in the A_340_ readings [41]. The reaction mixture consisted of 95 μl of assay buffer (100 mM Tris-Cl buffer, pH 6.5, 150 μM NADH, 300 mM of potassium-5-keto-D-gluconate) and 5 μl of purified His tagged SP 0320 protein (final concentration = 0.242 mg/ml). The A_340_ readings were recorded every 30 seconds for 10 minutes at 37 ^o^C using the Tecan GENius Plus plate reader. Potassium-5-keto-D-gluconate was purchased from Sigma.

### Biochemical assay of SP 0675 (D-Glucose dehydrogenase, EC 1.1.1.47) (S1 Fig) [42]

According to the PUMA 2 database, SP 0675 was predicted to function as a glucose dehydrogenase. Glucose dehydrogenase catalyzes the oxidation of D-glucose in the presence of either NAD(P)^+^ or NAD^+^ to form D-glucono-1, 5-lactone. The NAD(P)H or NADH formed can be monitored by an increase in the A_340_ readings. The reaction mixture consisted of 13 mM Tris-Cl buffer, pH 8.0, 0.5 mM NADP^+^ or NAD^+^ (Sigma), 0.1% bovine serum albumin (BSA), 0.5% Triton X 100, and 50 mM D-glucose. After 15 minute incubation at 37°C, 0.034 mg/ml of purified His tagged SP 0675 protein was added and the A_340_ readings were monitored every 30 seconds for 15 minutes at 37°C using the Tecan GENius Plus plate reader. In order to check for substrate specificity of SP 0675, a similar assay was done using glucose 6-phosphate (Sigma) instead of D-glucose as the substrate.

### *In vitro* glyceraldehyde 3-phosphate dehydrogenase (GAPDH) activity assay [43]

**Fig 2:**
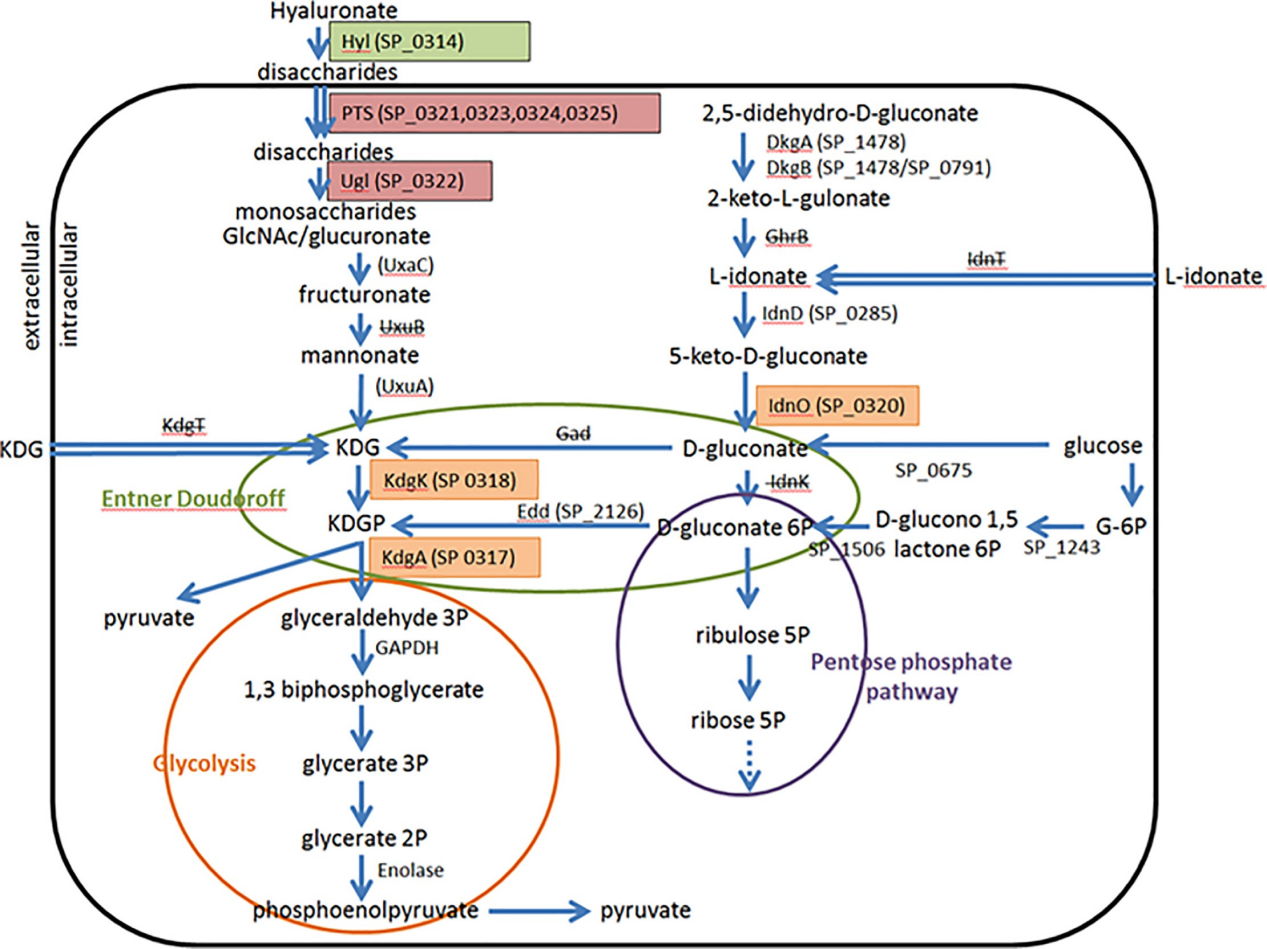
Proposed metabolic pathways of hyaluronate and L-idonate in *S*. *pneumoniae*. Solid arrows indicate the directions of metabolic processes. Dotted arrow shown in the pentose phosphate pathway (purple circle) indicates further reactions down the pathway. Double arrows indicate transports of molecules. Proteins whose names have been striked through indicate that homologs could not be identified among *S*. *pneumonia* strains. To correspond to Fig 1, candidate gene products in our study (SP 0317, SP 0318, SP 0320), the PTS transport system (*SP 0321*, *SP 0323*, *SP 0324*, *SP 0325*) along with glucuronyl hydrolase (*SP 0322*), and the hyaluronate lyase (Hyl) protein have been highlighted in orange, pink, and green, respectively. We postulate that SP 0675 functions as a glucose dehydrogenase, catalyzing the conversion of D-glucose to D-gluconate. In *S*. *pneumoniae*, KDG can be derived from two sources: mannonate and D-gluconate, via catabolism of hyaluronate (a glycosaminoglycan) and L-idonate, respectively. Catabolism of hyaluronate is initiated by a hyaluronate lyase (Hyl, encoded by *SP 0314*, located downstream to the *kdg* region). Hyl cleaves hyaluronate into disaccharides [55], which enter bacterial cell via a PTS transport system (encoded by *SP 0321*, *SP 0323*, *SP 0324*, *SP 0325*, upstream to the *kdg* region) [55, 67]. Once inside the cell, the disaccharides are further broken down into monosaccharides (D-glucuronate and N-acetyl-D-glucosamine) through glucuronyl hydrolase (Ugl), encoded by *SP 0322* [55, 68]. It is likely that the monosaccharide D-glucuronate is metabolized to KDG in *S*. *pneumoniae* via the enzymatic actions carried out by glucuronate isomerase (UxaC), fructuronate reductase (UxuB) and mannonate dehydratase (UxuA) as described in *E*. *coli* [65, 69]. Although the homolog of *E*. *coli’s* UxuB, which catalyzes the conversion of fructuronate to mannonate, could not be identified in *S*. *pneumoniae* (with mannitol-1-phosphate 5-dehydrogenase [SP 0397] being the closest homolog, sharing 25% amino acid identity), it is possible that an uncharacterized enzyme or an enzyme with multiple functions could carry out this process. Interestingly, the homologs of UxaC and UxuA of *E*. *coli* (which respectively catalyze the conversions of glucuronate to fructuronate and mannonate to KDG) were only identified in the single *S*. *pneumoniae* strain, 70585 (their names are respectively SP70585_ 2238 and SP70585_ 2237). In addition to the degradation of hyaluronate, which yields mannonate as a precursor molecule of KDG, metabolism of L-idonate and its subsequent conversion to D-gluconate is an alternative source of KDG in *S*. *pneumoniae*. Metabolism of L-idonate to D-gluconate occurs via two enzymatic processes: 1) the conversion of L-idonate to 5KG, carried out by L-idonate 5 dehydrogenase (IdnD) (encoded by *SP 0285*); and 2) conversion of 5KG to D-gluconate by IdnO (encoded by one of the candidate genes in our study: *SP 0320*) [41]. Although it has been shown in other organisms that KDG could be derived from other sources, such as the hexuronate galacturonate and plant pectin [70–72], these are less likely to be relevant in *S*. *pneumoniae* as none of the homologs required for the metabolism of galacturonate and pectin could be identified in *S*. *pneumoniae*. Metabolism of KDG to G3P via the Entner-Doudoroff pathway (shown in green) requires the enzymatic actions of KdgK and KdgA, which are respectively encoded by two of the genes in our study: *SP 0318* and *SP 0317*. KdgK and KdgA convert KDG to KDGP and KDGP to G3P and pyruvate, respectively. G3P produced could subsequently enter the latter steps of glycolysis (circled in orange) to yield further pyruvate and phosphoenolpyruvate. Abbreviations in the diagram: Hyl, hyaluronidase; PTS, phosphate transport system; Ugl, glucuronyl hydrolase; UxaC, glucuronate isomerase; UxuB, fructuronate reductase; UxuA, mannonate dehydratase; KDG, 2-keto-3-deoxygluconate; KDGP, 2-keto-3-deoxygluconate 6-phosphate; GAPDH, glyceraldehyde 3-phosphate dehydrogenase; IdnD, L-idonate 5 dehydrogenase; IdnO, 5-keto-D-gluconate-5-reductase; IdnK, D-gluconate kinase; Gad, gluconate dehydratase; Edd, phosphogluconate dehydratase; G-6P, glucose 6-phosphate.

We adopted a previously described protocol to determine the difference in cell membrane activities of GAPDH between mutant and wild type strains [43]. The GAPDH activity assay is based on the conversion of G3P to 1,3 biphosphoglycerate in the presence of inorganic phosphate (Fig 2), and the subsequent reduction of NAD^+^ to NADH resulting in an increase in the absorbance readings at 340 nm. Membrane protein lysates were extracted from overnight cultures by ultrasonication and ultracentrifugation at 50,000 x g for 90 minutes. The reaction mixture consisted of 10 mM NAD^+^, 3.5 mg/ml G3P solution (stock concentration at 50 mg/ml), 0.1 mg/ml membrane protein lysate, and the volume was adjusted to 0.1 ml with GAPDH assay buffer (40 mM Triethanolamine, 50 mM Na_2_HPO_4_, 5 mM EDTA, pH 8.6). The absorbance readings at 340 nm were monitored every 30 seconds for 30 minutes at 37°C using the Tecan GENius Plus plate reader.

## Results

### Genetic organization of the *pneumococcal kdg* region

SP 0320 and SP 0675 from the *S*. *pneumoniae* TIGR4 genome were identified as the two proteins with the highest homology to CsgA of *M*. *xanthus* (23 and 26% respectively). Since CsgA is implicated in biofilm formation in *M*. *xanthus* [21–23], gene deletions (knocokouts) of SP 0320 and SP 0675 were created to test this hypothesis that they might have a similar function. The genes *SP 0317*, *SP 0318*, and *SP 0319* are genes adjacent to *SP 0320*, and all four appear to be within a single operon and thus, potentially functionally linked to *SP 0320* (Fig 1). For convenience, we shall refer to the *SP 0317*, *SP 0318*, *SP 0319* and *SP 0320* genes collectively as the *kdg* region as we bioinformatically annotated them as being involved in ketogluconate metabolism with some of the proteins likely part of the Entner-Doudoroff pathway, which is an alternative pathway to glycolysis in the catabolism of hexose to pyruvate [44, 45] (Fig 2). Genes involved in the *kdg* region have been implicated in virulence in a number of bacteria [46, 47]. In the *Streptococcus dysgalactiae subsp*. *equisimilis*, upregulation of two of the Entner-Doudoroff pathway genes (*kdgK*, *kdgA*) were observed following four hours post infection in a murine infection model [46]. For this reason, we postulate that the *kdg* region of *S*. *pneumoniae* may play an important role in the pathogenesis of disease in this organism.

We annotated the functions of SP 0317, SP 0318, SP 0319, and SP 0320 as, respectively, 2-keto-3-deoxy-6-phospho-D-gluconate aldolase (KdgA), 2-keto-3-deoxy gluconate kinase (KdgK), ribose 5-phosphate isomerase, and 5-keto-D-gluconate-5-reductase (IdnO) (Fig 2). KdgA, KdgK, ribose 5-phosphate isomerase and IdnO are responsible for the conversions of KDGP to G3P and pyruvate, KDG to KDGP, ribulose 5-phosphate to ribose 5-phosphate, and 5 keto-D-gluconate (5KG) to D-gluconate, respectively (Fig 2) [39–41, 48, 49]. KdgA and KdgK are part of the Entner-Doudoroff pathway [50, 51]; and IdnO is involved in the catabolism of ketogluconate, a precursor step to the Entner-Doudoroff pathway (Fig 2) [39]. Although SP 0319 has been postulated to function as a ribose 5-phosphate isomerase [52], the exact pathway it is involved in is unclear at present.

SP 0675 showed homology to short chain dehydrogenases through a database search, with more detailed searches revealing that it was likely a glucose dehydrogenase (GD) based on its possession of five of six conserved GD motifs (S1 Table). Biochemical characterization of the expressed recombinant protein confirmed this supposition.

### Construction of mutant strains

Insertional-deletion mutant strains were created for the GD gene and each of the KDG genes to assess whether disruption of these candidate genes would have an effect on various phenotypes (e.g. growth rate in THB, biofilm formation, and virulence *in vivo*). The *SP 0320* and *SP 0675* single mutants as well as the *SP 0320*:*0675* double mutant were created in the serotype 23 strain BS72 [25] background (annotation based on the TIGR4 sequence). Mutations were also created in the *SP 0317*, *SP 0318* and *SP 0319* genes as they are likely transcribed as part of a single operon with *SP 0320*. Complemented *SP 0317*, *SP 0320* and *SP 0675* mutant strains were constructed to assess the role these genes play in virulence (Fig 1 and S2 Table).

### Growth of the mutant strains in THB

**Fig 3:**
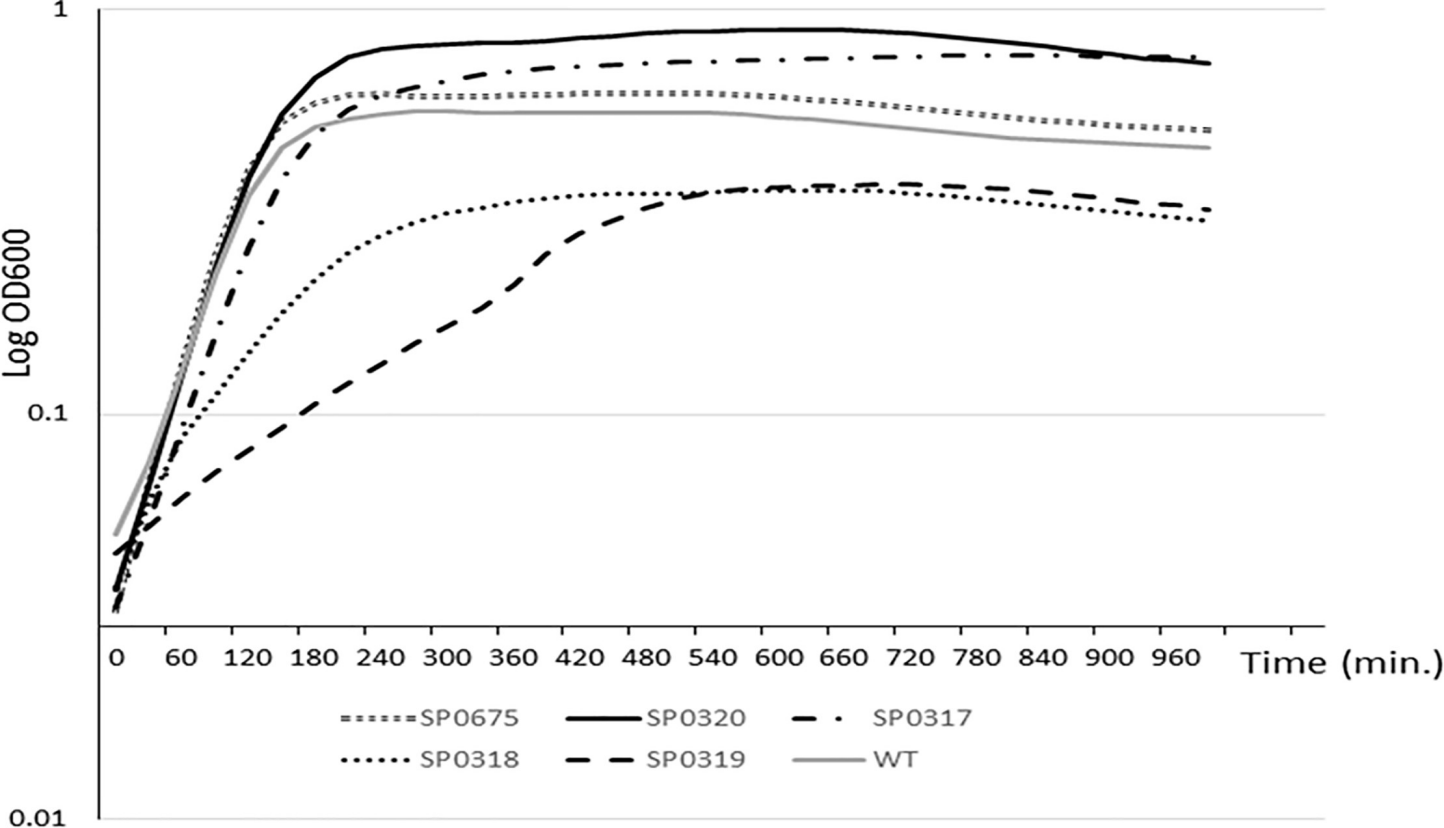
Growth curve of the wild type and mutant strains from three representative experiments.

We assessed whether mutations in *SP 0317*, *SP 0318*, *SP 0319*, *SP 0320* and *SP 0675* had an effect on the growth rate of bacteria in THB compared to the wild type (WT) strain. Three independent experiments for each mutant:WT pair showed that the *SP 0317*, *SP 0320* and *SP 0675* knockouts had slightly shorter doubling times 0.67±0.28, 0.69±0.3 and 0.67±0.28, respectively, than the WT strain. The *SP 0319* knocokout showed a much longer generation time (1.85±0.6) than the WT strain; and the *SP 0318* knockout initially showed similar growth rates (0.9±0.2) to the WT strain however, it doubling time significantly increased after first hour (Fig 3). Furthermore, the maximal cell densities of the cultures was also different. Mutants *SP 0317*, *SP 0320* showed *OD600* higher (1.4±0.3 and 1.6±0.4, respectively) while *SP 0675* mutant showed cell density similar to the WT strain (1.12±0.13). Both the *SP 0318 and SP 0319* knockouts showed much lower maximal cell densities than the WT strain (0.6±0.07 and 0.62±0.05 respectively) (Fig 3).

### Polysaccharide tests

**Fig 4:**
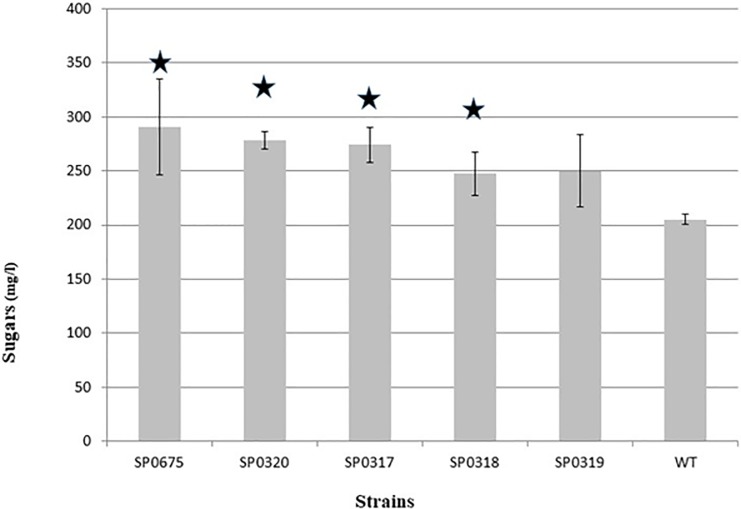
Total sugar content (mg/L, glucose equivalent) in the WT and mutant strains. Statistical significance compared to the wild type group are indicated by asterisks.

The capsular polysaccharide has been identified as the main virulence factor of *S*. *pneumoniae* [53]. Pneumococcus has evolved by diversifying its capsule, and up to 90 different capsular types synthesizing polysaccharides with different immunological properties and chemical structures have been described [54]. Since candidate genes in our study are postulated to have functions relating to sugar metabolism, we assessed whether mutations in *SP 0317*, *SP 0318*, *SP 0319*, *SP 0320* and *SP 0675* could have an effect on the amount of cellular polysaccharides. All mutant strains produced more polysaccharide (250–290 mg/L) than the WT strain (200mg/L) (Fig 4). All the differences except for *SP 0319* were statistically significant (P> 0.05 Student’s t-test).

### Effect of SP 0675 and SP0320 mutations on *regR* and *hyl* genes expression

**Fig 5:**
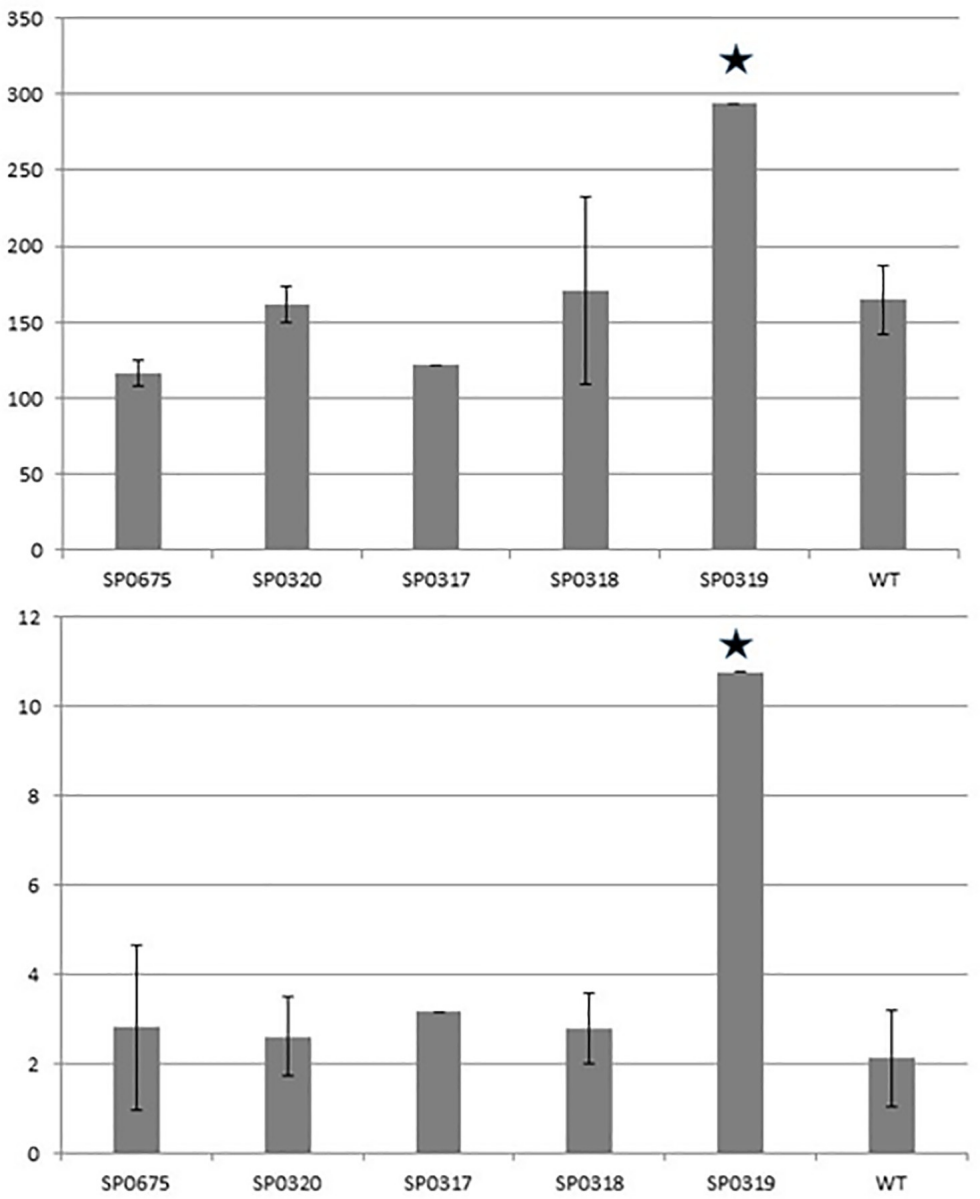
**Expression levels of the *regR* (upper) and *hyl* genes (lower graph) in SP 0317, SP 0318, SP 0319, SP 0320, SP 0675 and the WT strains shown in arbitrary units in relation to *gyrB* gene expression.** Data represents average of three biological replicates. The data points for SP 0317 and SP 0319 were almost identical, hence the SD bars are negligible. Statistical significance compared to the wild type group are indicated by asterisks.

The Kdg region on the pneumococcal chromosome is flanked on one side by *hyl* (*SP 0314*) and on the other by *regR* (*SP 0330*) (Fig 1) [55, 56]. We hypothesize that the *kdg* region of *S*. *pneumoniae* is under the control of RegR, and byproducts of the Kdg region might regulate expression of *regR* and subsequently the *hyl* gene. To test these hypotheses, we ran a quantitative PCR reaction using the Digital Droplet PCR method (Bio-Rad). We designed primers and probes specific for the *regR* and *hyl* genes. Gene *gyrB* was used as an internal control to normalize expression levels between strains and experiments. Results showed that expression of both the *regR* and *hyl* genes in SP0675, SP0320, SP0317 and SP0318 mutants was slightly lower or similar to those in the WT strain (Fig 5) and not statistically significant (P<0.05 Students t-test). Expression levels in the SP0319 mutant were significantly higher (1.8 and 5 fold for the *regR* and *hyl*, respectively; P>0.05 Students t-test) than in the WT strain. To compare, in the *regR* mutant strain activity of the *hyl* gene was 14.4 times higher than in the WT strain (S4 Fig).

### Characterization of the functions of SP 0317, SP 0318, SP 0320 and SP 0675 through biochemical assays

N-terminal His-tagged SP 0317, SP 0318, SP 0319, SP 0320 and SP 0675 proteins were overexpressed and purified using pET 302/NT-His and BL21 DE3 *E*. *coli* overexpression system (S2 Fig). The postulated functions of SP 0317, SP 0318 and SP 0320 were respectively KdgA, KdgK, and IdnO, and these were confirmed using biochemical assays (Table 1, S1 Fig).

**Table 1:** Biochemical activities of SP 0317, SP 0318, SP 0320 and SP 0675 purified proteins.

Protein names	Protein functions	Biochemical activities
SP 0317	2-keto-3-deoxy-6-phospho-D-gluconate aldolase	Formation of NAD+ results in a decrease in A340 readings
SP 0318	2-keto-3-deoxy-glucono kinase	Formation of NADH results in an increase in A340 readings
SP 0320	5-keto-D-gluconate-5-reductase	Formation of NAD+ results in a decrease in A340 readings
SP 0675	D-glucose dehydrogenase	Formation of NADH results in an increase in A340 readings

Biochemical activities are shown in Supplementary S1 Fig.

According to the PUMA 2 database, SP 0675 could function as an oxidoreductase or a glucose dehydrogenase. A typical glucose dehydrogenase catalyzes the oxidation of D-glucose in the presence of NAD(P)^+^ or NAD^+^ to form D-glucono-1,5-lactone. Our biochemical assay confirmed that SP 0675 functions as a glucose dehydrogenase, utilizing D-glucose as the substrate (S1 Fig); and thus, we hypothesize that SP 0675 is involved in the conversion of D-glucose to D-gluconate, similar to that seen in *S*. *solfataricus* (Fig 2) [57]. According to the Prints database (http://bioinf.man.ac.uk/dbbrowser/PRINTS/index.php) that examines conserved motifs of proteins, we were able to identify five out of six motifs of the glucose ribitol dehydrogenase family in SP 0675 (S1 Table). The glucose ribitol dehydrogenase protein family encompasses closely related dehydrogenases, dehydroxylases and reductases.

### Extent of biofilm formation by the wild type and mutant strains

Since SP 0320 and SP 0675 have some homology to CsgA of *M*. *xanthus*, we evaluated their role in biofilm formation. However, we were unable to observe a significant difference in the biofilm forming phenotype in the *SP 0320* and *SP 0675* knockout strains compared to the wild type strain using the chinchilla model of otitis media (S3 Fig).

Furthermore, biofilm formation studied using flow cells visualized by live/dead staining (Molecular Probes) on a confocal laser scanning microscope also showed no significant difference in the biomass and roughness coefficients of the biofilms formed by the wild type and mutant strains, indicating that SP 0320 and SP 0675 are most likely not involved in biofilm formation.

### Comparative pathogenicity of the wild type BS72 strain and mutants of the CsgA homologs

The chinchilla model of OMID was used to determine the differences in virulence between the wild type strain BS72 and the various mutant constructs detailed above. Animals were kept in pathogen free conditions, had 24 hour access to food and water and 14hr/10hr light/dark cycling. Animals were followed for ten days, or until death or moribundity at which point they were euthanized. Animals with a moribundity score of 4 (S7 Table) were euthanized. Cohorts of ten to fifteen animals were inoculated bilaterally using a transbullar approach with 10^3^ bacteria in a 100 μl volume for all wild type and mutant evaluations; a sham inoculated cohort was similarly inoculated but with no bacterial component and monitored for ten days. Animals were evaluated using a video otoscope on a daily basis and local disease severity was determined by two board-certified otolaryngologists using the scoring system described in Methods. Systemic signs were evaluated periodically around the clock by the animal husbandry staff and upon moribundity, the laboratory staff was called in to euthanize the animals. All surviving animals were euthanized at the completion of the ten days evaluation period.

**Fig 6:**
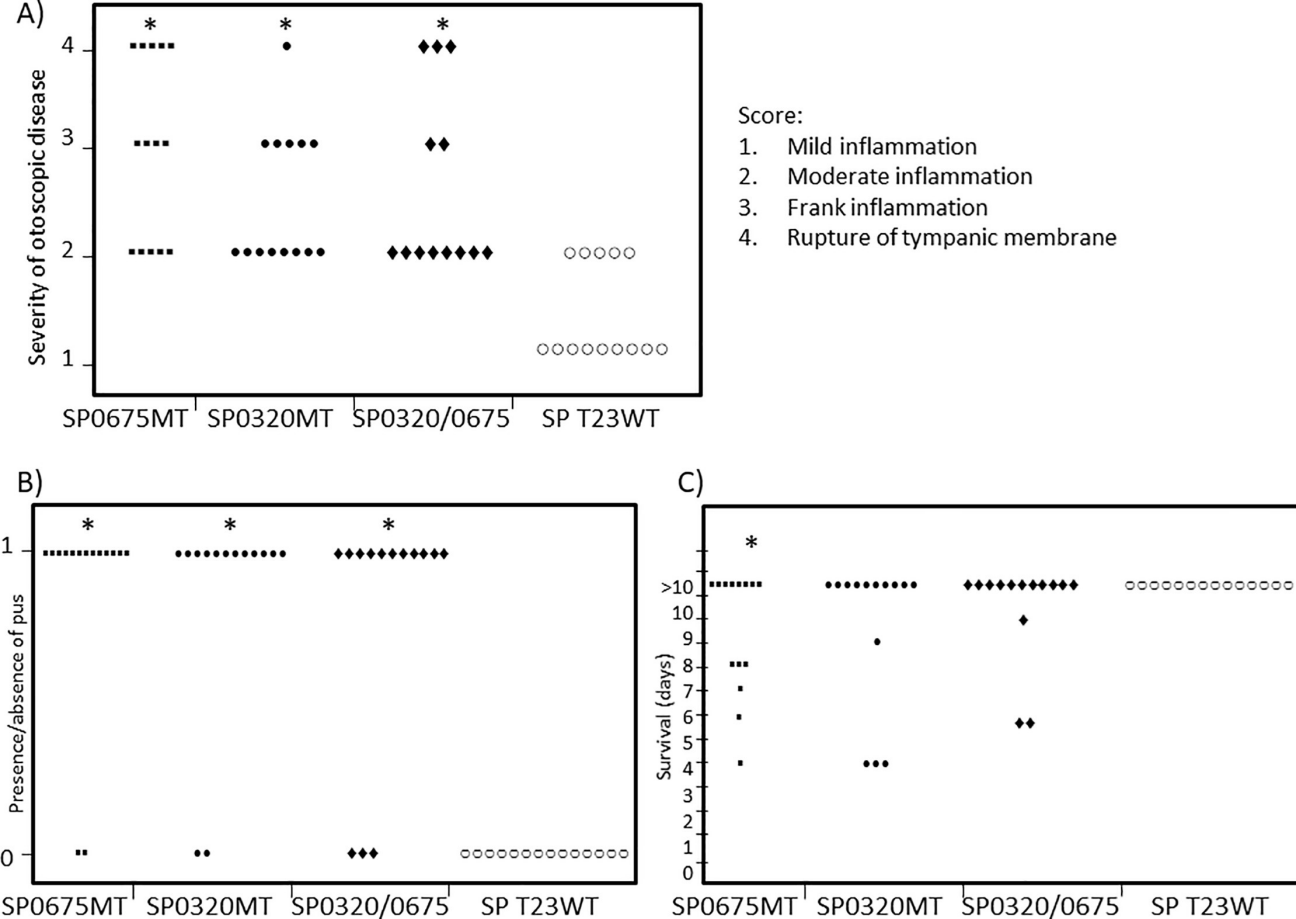
**Animal test results; (A) Maximum otological scores of animal ears inoculated with the wild type, *SP 0320*, *SP 0675* and the *SP 0320*:*0675* double mutant strains.** Chinchillas injected transbullarly with the *SP 0320*, *SP 0675* or the *SP 0320*:*0675* double mutant showed greater severity of otoscopic disease compared to the wild type inoculated animals following a ten day study. The Kruskal-Wallis rank sum test gave a *p-value* of 1.47 x 10−^5^. Statistical significance compared to the wild type group is indicated by asterisks. Scoring was done by board-certified otolaryngologists. (B) The presence or absence of pus formation in animal ears inoculated with the wild type and *SP 0320*, *SP 0675* or the *SP 0320*:*0675* double mutant strains. No pus was recovered from the middle ears of wild type inoculated animals. Pus was recovered in the ear effusion of nearly 86% of animals inoculated with either the *SP 0320* or the *SP 0675* mutant, and from 77% of animals inoculated with the *SP 0320*:*0675* double mutant (*P* = 1.39 x 10^−7^, 1.39 x 10^−7^ and 3.39 x 10^−5^ respectively using the Fisher’s exact test to compare each group to the wild type strain). Statistical significance compared to the wild type group is indicated by asterisks. (C) Survival and mortality of animals inoculated with the wild type and *SP 0320*, *SP 0675* or the *SP 0320*:*0675* double mutant strains. In the *SP 0320*:*0675* double mutant inoculated cohort, one animal died within less than 24 hours due to unknown cause and was eliminated from the analyses. All animals survived beyond the 10^th^ day in the wild type group. *P-values* are 0.016, 0.098 and 0.098 respectively using the Fisher’s exact test to compare each group against the control. Statistical significance compared to the wild type group is indicated by asterisks.

The cohorts of chinchillas inoculated with the *SP 0320* and *SP 0675* gene mutants, and the double *SP 0320*:*0675* mutant showed vastly greater otoscopic disease severity compared to those animals injected with the wild type strain (Fig 6A, *p* = 1.47 x 10^−5^, the Kruskal-Wallis rank sum test). Upon postmortem dissection of the wild type and sham-inoculated animals on the tenth day, no pus was observed from any of the ears (Fig 6B). In contrast, pus was recovered from the middle-ear effusions of 86% of the animals inoculated with either the *SP 0320* or the *SP 0675* mutant, and from 77% of the animals inoculated with the *SP 0320*:*0675* double mutant (Fig 4B, *p* = 1.39 x 10^−7^, 1.39 x 10^−7^ and 3.39 x 10^−5^, respectively when compared to control, Fisher’s exact test). Twenty three percent and 35% of the ears inoculated with the double mutant and the *SP 0675*, respectively, showed tympanic membrane rupture (Fig 6A). Examination of otoscopic changes of animals inoculated with the wild type, *SP 0320*, *SP 0675* and the *SP 0320*:*0675* double mutant strains were performed and recorded on a daily basis (S3 Table).

**Fig 7:**
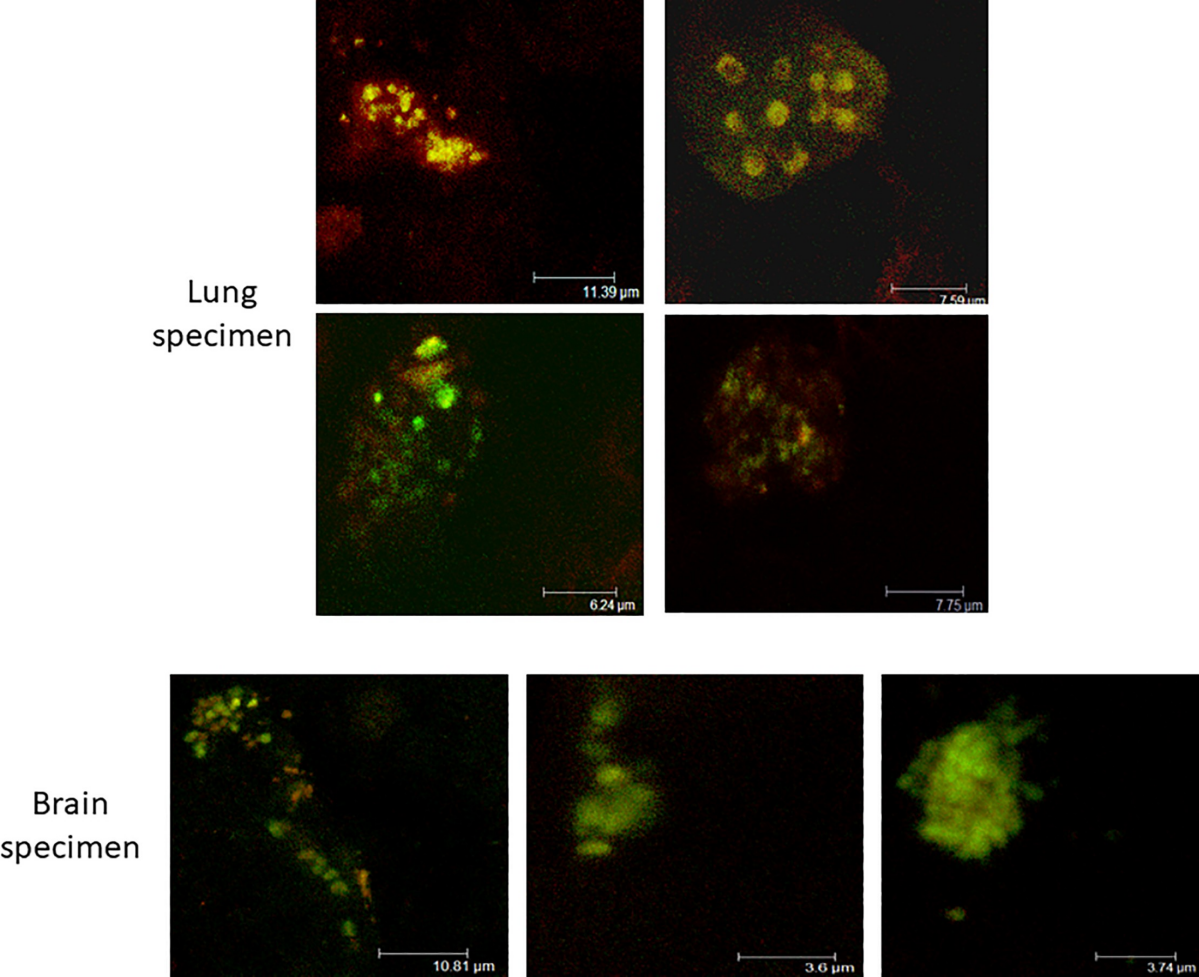
FISH of chinchilla lung and brain necropsy specimens following transbullar infection with the *SP 0320* mutant strain. 16S rRNA gene specific probe labeled with pneumococcal specific Cy3 and FAM was hybridized in the presence of formamide to the tissue samples. Green = pneumococcal specific; Red = eubacterial generic; yellow = overlay of the two dyes. The animal died three days post infection.

Evaluation of systemic differences in disease severity based on mortality demonstrated that 43% of animals inoculated with the *SP 0675* mutant strain, 29% of animals inoculated with the *SP 0320* mutant strain, and 23% of the animals inoculated with the *SP 0320*:*0675* double mutant died during the ten day evaluation period, whereas none of the wild type infected animals died (Fig 6C, *P* = 0.016, 0.098 and 0.098 respectively, Fisher’s exact test). There was no association between the mortality rate and the severity of ear diseases in these strains (*P* = 0.28, Fisher’s exact test). Inoculated bacteria could be recovered by plating from the middle-ear effusions of most of the animals inoculated with the *SP 0320*, *SP 0675* single mutants and the double mutant, as well as from the lung and/or the brain tissues of these animals recovered at autopsy (Fig 7). In animals inoculated with the wild type strain, bacteria could be recovered from the middle-ear effusion of six animals, but were never recovered from the brain or the lung. The otoscopic virulence and the mortality rate induced by infection with the *SP 0320*:*0675* double mutant was comparable to either of the single mutants (*P* = 0.44 and *P* = 0.77, respectively, Fisher’s exact test), which indicates a lack of synergistic effect due to individual mutations.

### Comparative pathogenicity of the wild type BS72 strain and the KDG gene mutants

Insertional deletion of *SP 0317* gene also resulted in a vastly increased mortality rate compared to the wild type (*P* = 0.0047, Fisher’s exact test), although the otoscopic virulence was comparable to the wild type strain. Surprisingly, the virulence of *SP 0318* and *SP 0319* knockouts was comparable to the wild type. The degree of virulence of the complemented *SP 0317*, *SP 0320* and *SP 0675* mutant strains was comparable to the wild type, confirming that the absence of these genes triggers increased virulence.

### *In vitro* GAPDH assay from membrane protein extract

We hypothesize that accumulation of GAPDH would occur in the *SP 0317* mutant strain (Fig 2). Since GAPDH could be exported extracellularly to bind host zymogens such as fibrinogen [58–62], it is possible that the increase in mortality of chinchillas inoculated with the *SP 0317* mutant strain compared to the wild type strain was due to the effect of extracellular accumulation of GAPDH in this mutant strain.

We used a previously described GAPDH assay [43] to help us determine the differences in cell membrane activities of GAPDH between mutant and wild type strains. The GAPDH activity assay is based on the conversion of G3P to 1,3 biphosphoglycerate in the presence of inorganic phosphate (Fig 2), and the subsequent reduction of NAD^+^ to NADH, resulting in an increase in absorbance reading at 340 nm.

**Fig 8:**
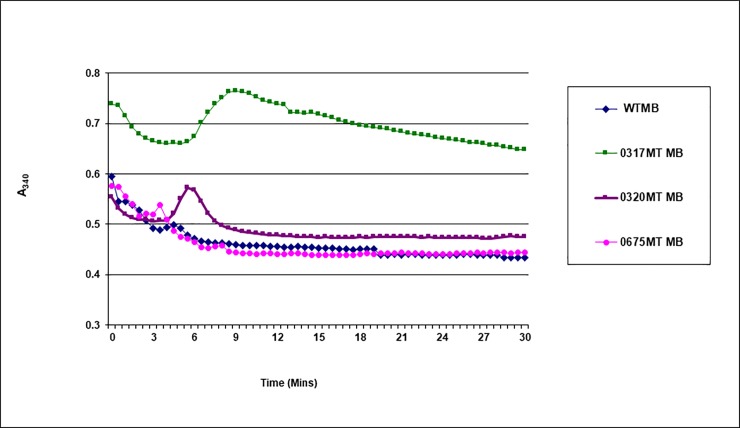
GAPDH activity of membrane protein lysates. The membrane protein lysate of the *SP 0317* mutant strain (shown in green) had a 1.7 fold greater activity of GAPDH compared to the wild type strain (shown in blue); while the membrane protein lysates of the *SP 0320* (shown in purple) and *SP 0675* (shown in pink) mutant strains respectively had a 1.1 and 1.2 fold greater activities compared to the wild type strain. Data show the representative from three independent experiments.

Our results indicated that the membrane protein extract of the *SP 0317* mutant strain had a 1.7 fold greater GAPDH activity compared to the wild type strain, whereas the membrane protein extract of the *SP 0320* and *SP 0675* mutant strains were similar to the wild type strain (1.1 and 1.2 fold greater activities compared to the wild type strain, respectively) (Fig 8).

## Discussion

*S*. *pneumoniae* is frequently recovered as a carriage isolate from the human nasopharynx, but it is also a major respiratory pathogen that can cause otitis media, bacteremia and meningitis. One of the crucial prerequisites for a pathogen to establish an infection within its host is the ability to invade cutaneous and mucosal surfaces and enter deep into the tissue sites.

In this study, we investigated a number of genes (*SP 0317*, *SP 0320*, *SP 0675*) whose disruption, and hence loss of functions to the proteins they encode, significantly increases the virulence of *S*. *pneumoniae in vivo* using the chinchilla OMID model. The genes *SP 0320* and *SP 0675* were identified because the proteins they encoded had the highest homology (23% and 26% identity, respectively) within the pneumococcal supragenome [25] to the CsgA protein of *M*. *xanthus*, which plays an important role in biofilm formation. In spite of this homology, we were unable to demonstrate that the pneumococcal gene products played any significant role in biofilm formation using the chinchilla OMID study and a flow cell system. The accessible databases of SP 0320 and SP 0675 indicated their possible involvement in sugar metabolism through ketogluconate metabolism (a precursor step to the Entner-Doudoroff pathway) (Fig 2) and glucose dehydrogenase, respectively. It is not unfamiliar that proteins involved in sugar metabolism could affect virulence of bacteria, as King and colleagues have demonstrated that pneumococcal proteins involved in sugar metabolism through deglycosylation are implicated in the physiology and virulence of the organism [63].

Based on multiple alignment searches, we tentatively identified SP 320 as the *idnO* gene, which is responsible for the conversion of 5KD to D-gluconate, a precursor step to the Entner-Doudoroff pathway (Fig 2). We also predicted that SP 0675 acted as a glucose dehydrogenase, involved in the conversion of D-glucose to D-gluconate, similar to that seen in *S*. *solfataricus* (Fig 2) [57]. Our biochemical studies of recombinantly expressed SP 0320 and SP 0675 proteins from *E*. *coli* confirmed these bioinformatically imputed functions.

In addition to our investigation of *SP 0320*, we included the rest of the genes in the same operon (*SP 0317*, *SP 0318* and *SP 0319*) in our study and bioinformatically annotated them as *kdg* genes involved in the Entner-Doudoroff pathway. SP 0317, SP 0318 and SP 0319 were found to be homologous to KdgA, KdgK, and ribose 5-phosphate isomerase, respectively. Although ribose 5-phosphate isomerase does not seem to play a role in the Entner-Doudoroff pathway, the other two enzymes do (Fig 2). Genes involved in KDG metabolism appear to be important for the development of virulence of bacteria *in vivo*, as upregulation of both *kdgK* and *kdgA* were observed in *Streptococcus dysgalactiae subsp*. *equisimilis* following four hour post infection in a murine model of infection [46]. Moreover, using the Tn-Seq technique, van Opijnen and colleagues demonstrated that mutations in *SP 0317* and *SP 0319* reduced colonization of *S*. *pneumoniae* and affected virulence of bacterium in a murine model of infection [64].

We have developed two, non-exclusive hypotheses based on our observation of the increased virulence in the *SP 0317* and *SP 0320* knockout mutants in the chinchilla model of OMID with regard to how the deletion of genes involved in the Entner-Doudoroff pathway and ketogluconate metabolism could result in increased virulence. In the first instance, we note that several enzymes in the glycolytic pathway (such as GAPDH and enolase) of the pneumococci and other streptococci are bifunctional: in addition to their roles in glycolysis, they are also sometimes found on the cell surface where they are known to bind host zymogens such as fibrinogen [58–62]. The binding and activation of these host degradative enzymes by the glycolytic enzymes serves to imbue the bacteria with increased invasiveness as they can now degrade the intercellular matrix allowing bacterial penetration into deep tissue sites. The mechanisms by which these glycolytic enzymes are translocated to the cell surface are unknown, as are the signals that trigger the process. We hypothesize that a reduction in carbon flow through glycolysis (triggered by mutation in *SP 0317*) is part of the signaling process that results in increased translocation of the aforementioned bifunctional enzymes (such as GAPDH) to the cell surface. In this scenario, the loss of function associated with the *SP 0317* deletion mutant would result in the bacterium continually sensing a decrease in carbon flow through the glycolytic pathway, which to compensate for it maximally and continuously translocates the bifunctional enzyme (GAPDH) to the cell surface, resulting in high levels of zymogen binding and invasiveness.

The second hypothesis comes from the fact that the *kdg* region of the pneumococcal chromosome is flanked on one side by *hyl* (*SP 0314*) and on the other by a PTS operon (*SP 0321*, *SP 0323*, *SP 0324*, *SP 0325*) and *RegR* (*SP 0330*) (Fig 1) [56, 65]. We hypothesize that the *kdg* region of *S*. *pneumoniae* is under the control of RegR, which belongs to the LacI/GalR regulator family, similar to the IdnR regulator of *E*. *coli*. The IdnR regulator of *E*. *coli* functions as a repressor protein that regulates the expression of genes involved in the L-idonate pathway [66]. In *E*. *coli*, both L-idonate and 5KG are inducers of the L-idonate pathway, likely through their interaction with IdnR [66]. Since accumulation of 5KG is expected to occur in the *SP 0320* mutant, and it has already been shown that RegR negatively regulates the expression of *hyl* [56], 5KG that would be accumulated in the *SP 0320* mutant strain could potentially act as an inducer to both the *kdg* and *hyl* operons, leading to increased hyaluronidase activity and conferring an increase in virulence of *S*. *pneumoniae in vivo* (as observed in our *SP 0320* knockout mutant). Although our data did not support this hypothesis it cannot be rejected as the experimental *in vitro* conditions are different from those the bacteria are exposed in vivo during infection.

We still think that both models could contribute to the increased virulence observed following infection with the knockout strains. We are currently performing experiments to validate both hypotheses.

## Supporting information

S1 Table SP 0675 contains five out of six conserved motifs of the glucose ribitol dehydrogenase family. According to the Prints database (SPRINT - http://130.88.97.239/dbbrowser/sprint/), the glucose ribitol dehydrogenase protein family contains six conserved motifs http://130.88.97.239/cgi-bin/dbbrowser/sprint/searchprintss.cgi?prints_accn=PR00081&display_opts=Prints&category=None&queryform=false&regexpr=off and SP 0675 contains five out of the six conserved motifs PR00081 (https://www.ebi.ac.uk/interpro/protein/A0A0H2UP37). This protein family consists of closely related dehydrogenases, dehydroxylases and reductases, and was initially derived from the alignment of eleven glucose and ribitol dehydrogenases.
(DOCX)

S2 Table Bacterial strains used in this study. (DOCX)

S3 Table Degree of otoscopic changes observed in the ears of chinchillas inoculated with the SP *0320*, *SP 0675* and the *SP 0320:0675* double mutant and WT strains. Score of 0, 1, 2, 3 and 4 respectively indicate no symptom, mild inflammation, moderate inflammation, frank purulence and rupture of tympanic membrane.
(DOCX)

S4 Table Accession numbers of candidate genes in our study. (DOCX)

S5 Table **Table A. Primers used for mutation and confirmation** (Restriction sites are in bold). **Table B. Primers used for making complemented mutants and for confirmation** (Restriction sites are in bold). **Table C. Primers used for amplifying *SP 0317, SP 0318, SP 0319, SP 0320* and *SP 0675* to construct over expression plasmids and for confirmation** (Restriction sites are in bold). **Table D. Primers and probes used for DDPCR**.
(DOCX)

S6 Table Plasmids and DNA templates used in this study. (DOCX)

S7 Table Otologic score for the chinchilla otitis media experiment. (DOCX)

S1 Fig Biochemical assays for the SP 0317, SP 0318, SP 0320 and SP 0675 purified proteins. Panel A: 2-keto-3-deoxy-6-phospho-D-gluconate aldolase reaction catalyzed by His- tagged SP 0317 protein (♦). Formation of NAD^+^ during this reaction results in a decrease in A_340_ readings. Negative control (minus SP 0317) did not show the aldolase reaction (■).
Panel B: 2-keto-3-deoxy-glucono kinase reaction catalyzed by His-tagged SP 0318 protein (♦). Formation of NADH during this reaction results in an increase in A_340_ readings. Negative control (minus SP 0318) did not show the kinase reaction (■).
Panel C: 5-keto-D-gluconate-5-reductase reaction catalyzed by His- tagged SP 0320 protein (♦). Formation of NAD^+^ during this reaction results in a decrease in A_340_ readings. Negative control (minus SP 0320) did not show the reductase reaction (■).
Panel D: NADP^+^-dependent (•) and NAD^+^-dependent (■) D-glucose dehydrogenase reaction catalyzed by His- tagged SP 0675 protein. Formation of NADH during this reaction results in an increase in the A_340_ readings. Negative control (minus SP 0675) did not show dehydrogenase reaction (▲). In the presence of glucose 6-phosphate as the substrate, dehydrogenase reaction did not take place (data not shown).
(TIF)

S2 Fig SDS PAGE of pure His tagged SP 0317, SP 0318, SP 0319, SP 0320 and SP 0675 proteins. The N-terminal His tagged proteins obtained were 97% pure. Molecular weights (including that of N-terminal 6 X His tag) are as follows: SP 0317: 24 kDa (lane 2), SP 0318: 38.85 kDa (lane 3), SP 0319: 25.2 kDa (lane 4), SP 0320: 30.5 kDa (lane 5), and SP 0675: 29.5 kDa (lane 6)
(TIF)

S3 Fig FISH of bullae of chinchillas inoculated with the *SP 0320* and *SP 0675* mutant and wild type strains. 16S rRNA gene specific probe labeled with pneumococcal specific Cy3 and FAM was hybridized in the presence of formamide to the tissue samples. Green = Sp specific; Red = eubacterial generic; yellow = overlay of the two dyes.
(TIF)

S4 Fig Expression levels of the *regR* (upper) and *hyl* genes (lower graph) in SP 0317, SP 0318, SP 0319, SP 0320, SP 0675, SP 0330 and the WT strains shown in arbitrary units in relation to *gyrB* gene expression.
(TIF)
