# Supplementary figures and images for: Deletion of genes involved in the ketogluconate metabolism, Entner-Doudoroff pathway, and glucose dehydrogenase increase local and invasive virulence phenotypes in Streptococcus pneumoniae
Source: PLoS One. 2019 Jan 8;14(1):e0209688. doi: 10.1371/journal.pone.0209688 (PMC6324787; doi:10.1371/journal.pone.0209688)

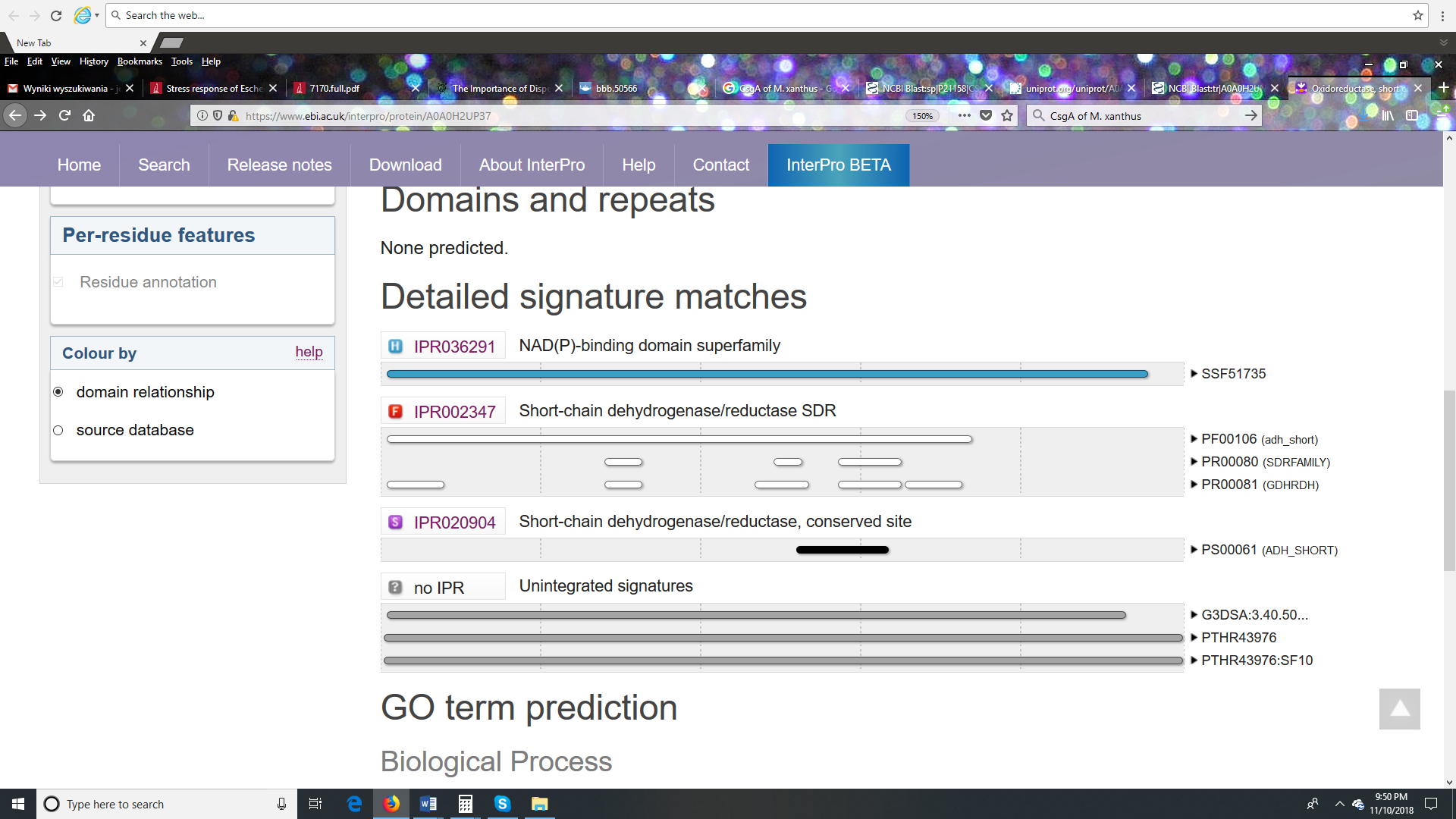


| Motif # | Motif length | Blast Identity | Blast Similarity |
| --- | --- | --- | --- |
| 1 | 18 | 14/18 (78%) | 17/18(94%) |
| 2 | 12 | 11/12(94%) | 12/12(100%) |
| 3 | 17 | 8/14(57%) | 9/14(64%) |
| 4 | 20 | 16/20(80%) | 20/20(100%) |
| 5 | 18 | 10/18(55%) | 16/18(89%) |
| 6 | 21 | Not present | Not present |

Supplement: S1 Table — According to the Prints database (SPRINT - http://130.88.97.239/dbbrowser/sprint/), the glucose ribitol dehydrogenase protein family contains six conserved motifs http://130.88.97.239/cgi-bin/dbbrowser/sprint/searchprintss.cgi?prints_accn=PR00081&display_opts=Prints&category=None&queryform=false®expr=off and SP 0675 contains five out of the six conserved motifs PR00081 (https://www.ebi.ac.uk/interpro/protein/A0A0H2UP37). This protein family consists of closely related dehydrogenases, dehydroxylases and reductases, and was initially derived from the alignment of eleven glucose and ribitol dehydrogenases. (DOCX) [file pone.0209688.s001.docx]

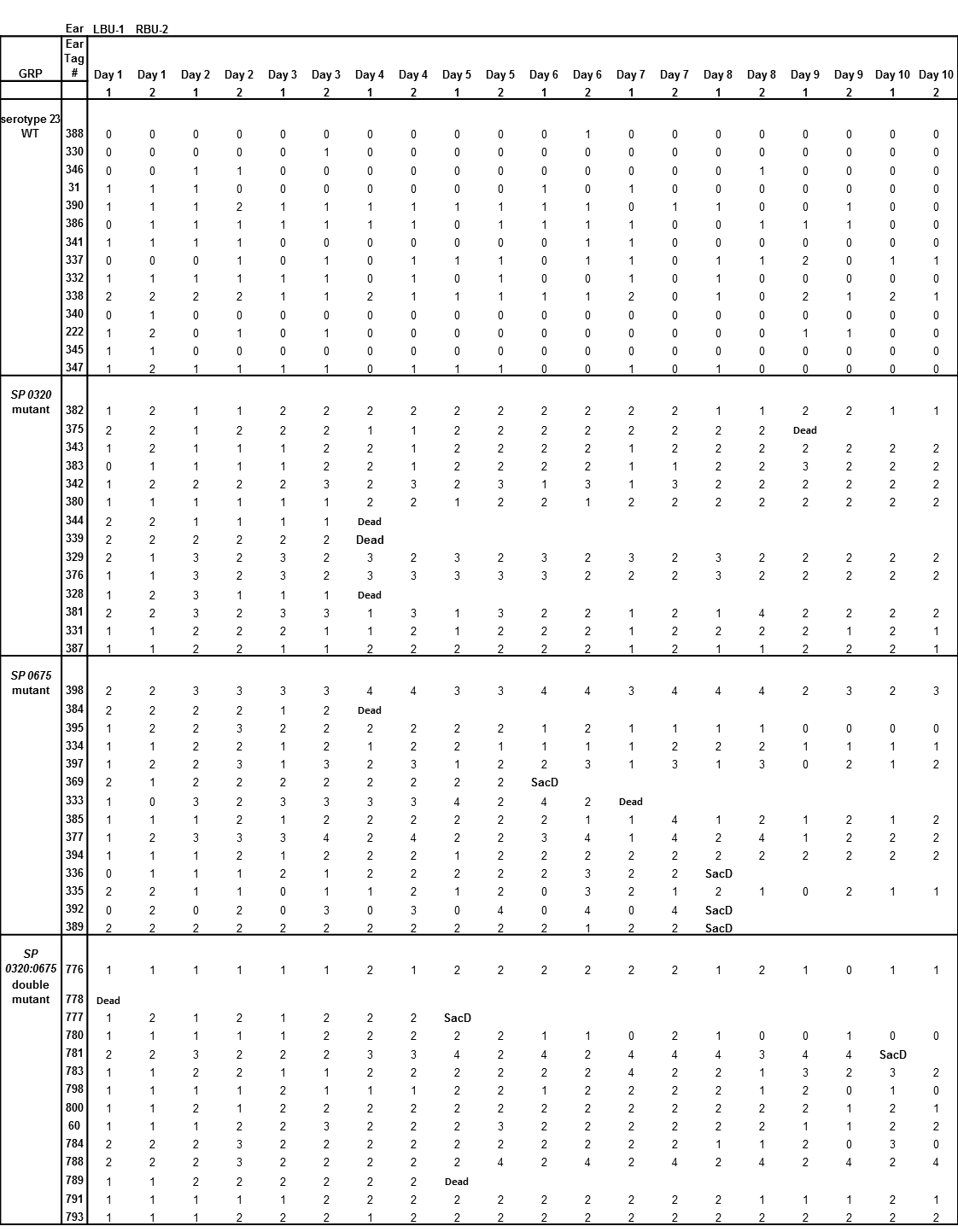

Supplement: S3 Table — Score of 0, 1, 2, 3 and 4 respectively indicate no symptom, mild inflammation, moderate inflammation, frank purulence and rupture of tympanic membrane. (DOCX) [file pone.0209688.s003.docx]

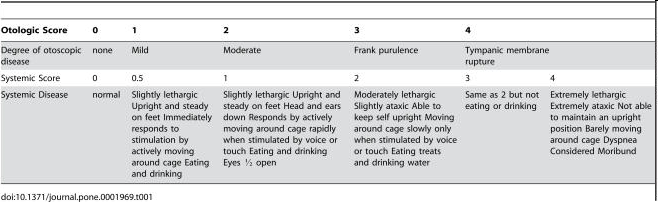

Supplement: S7 Table — (DOCX) [file pone.0209688.s007.docx]

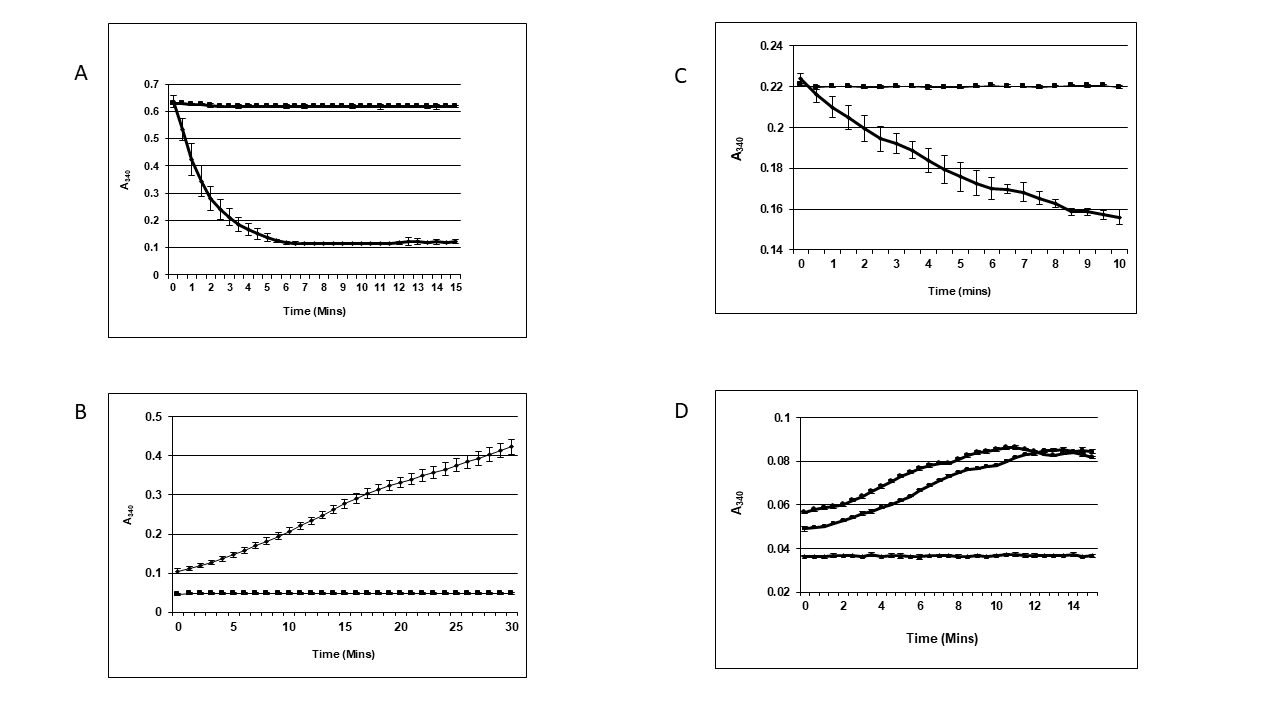

Supplement: S1 Fig — Panel A: 2-keto-3-deoxy-6-phospho-D-gluconate aldolase reaction catalyzed by His- tagged SP 0317 protein (♦). Formation of NAD+ during this reaction results in a decrease in A340 readings. Negative control (minus SP 0317) did not show the aldolase reaction (■). Panel B: 2-keto-3-deoxy-glucono kinase reaction catalyzed by His-tagged SP 0318 protein (♦). Formation of NADH during this reaction results in an increase in A340 readings. Negative control (minus SP 0318) did not show the kinase reaction (■). Panel C: 5-keto-D-gluconate-5-reductase reaction catalyzed by His- tagged SP 0320 protein (♦). Formation of NAD+ during this reaction results in a decrease in A340 readings. Negative control (minus SP 0320) did not show the reductase reaction (■). Panel D: NADP+-dependent (•) and NAD+-dependent (■) D-glucose dehydrogenase reaction catalyzed by His- tagged SP 0675 protein. Formation of NADH during this reaction results in an increase in the A340 readings. Negative control (minus SP 0675) did not show dehydrogenase reaction (▲). In the presence of glucose 6-phosphate as the substrate, dehydrogenase reaction did not take place (data not shown). (TIF) [file pone.0209688.s008.tif]

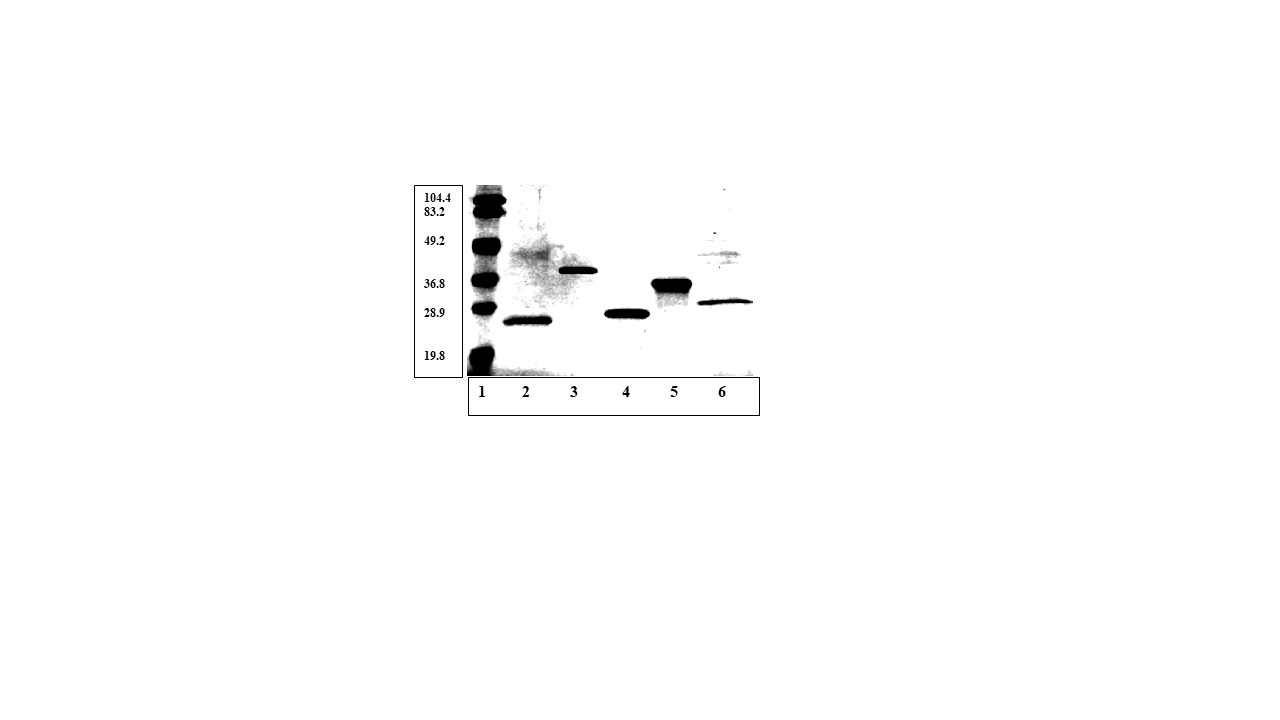

Supplement: S2 Fig — The N-terminal His tagged proteins obtained were 97% pure. Molecular weights (including that of N-terminal 6 X His tag) are as follows: SP 0317: 24 kDa (lane 2), SP 0318: 38.85 kDa (lane 3), SP 0319: 25.2 kDa (lane 4), SP 0320: 30.5 kDa (lane 5), and SP 0675: 29.5 kDa (lane 6) (TIF) [file pone.0209688.s009.tif]

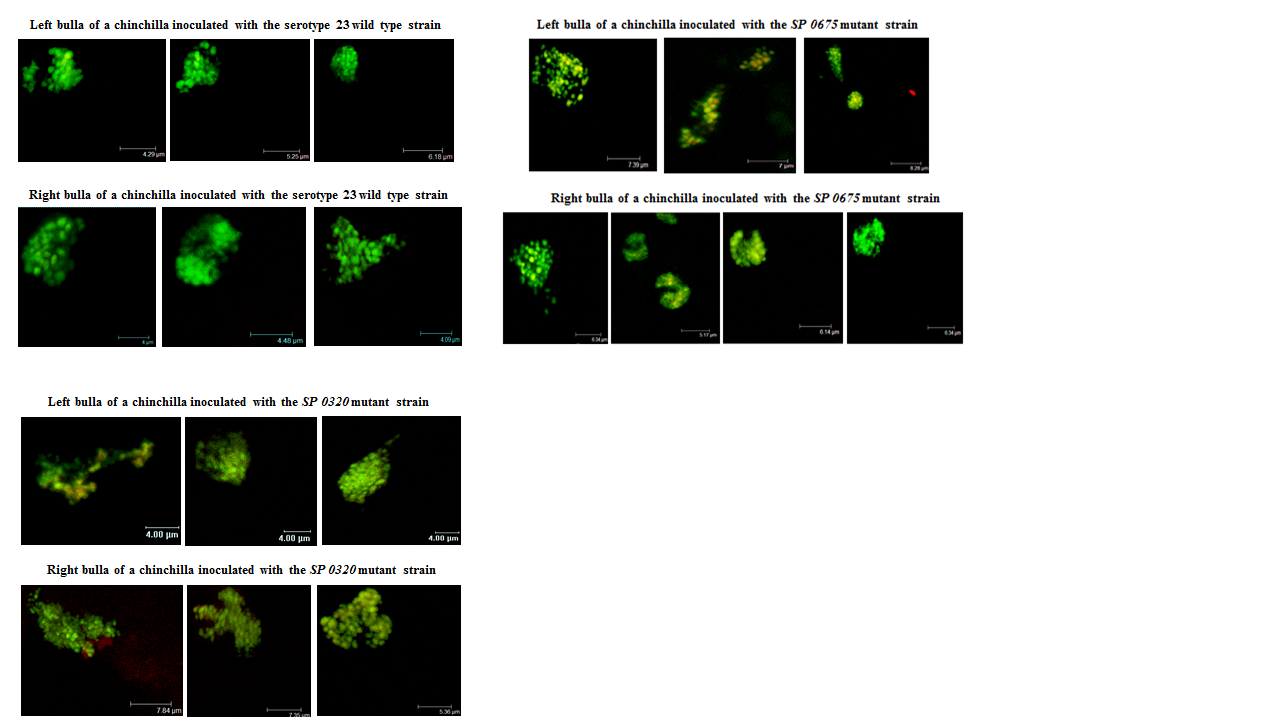

Supplement: S3 Fig — 16S rRNA gene specific probe labeled with pneumococcal specific Cy3 and FAM was hybridized in the presence of formamide to the tissue samples. Green = Sp specific; Red = eubacterial generic; yellow = overlay of the two dyes. (TIF) [file pone.0209688.s010.tif]

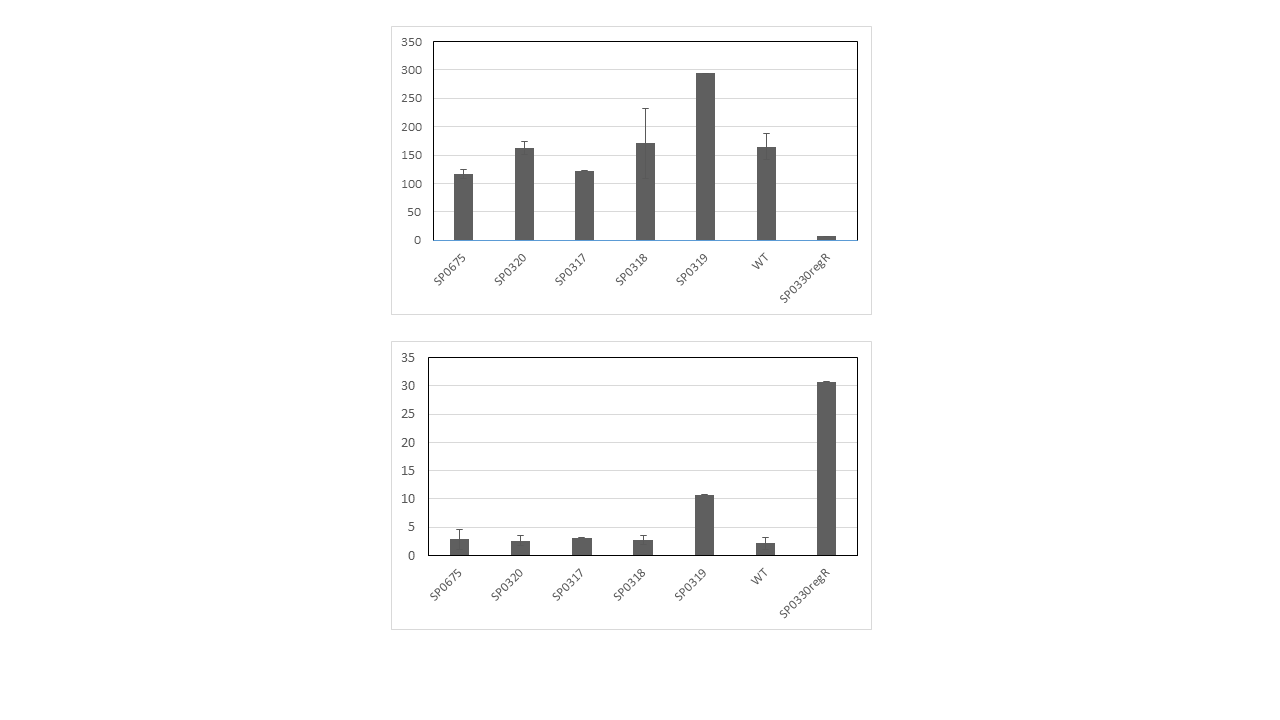

Supplement: S4 Fig — Expression levels of the regR (upper) and hyl genes (lower graph) in SP 0317, SP 0318, SP 0319, SP 0320, SP 0675, SP 0330 and the WT strains shown in arbitrary units in relation to gyrB gene expression. (TIF) [file pone.0209688.s011.tif]
