# Supplementary material for: Deletion of genes involved in the ketogluconate metabolism, Entner-Doudoroff pathway, and glucose dehydrogenase increase local and invasive virulence phenotypes in Streptococcus pneumoniae
Source: PLoS One. 2019 Jan 8;14(1):e0209688. doi: 10.1371/journal.pone.0209688 (PMC6324787; doi:10.1371/journal.pone.0209688)
Supplement: S2 Table — (DOCX) [file pone.0209688.s002.docx]

| **Strain name** | **Host name** | **Strain description** |
| --- | --- | --- |
| *S. pneumoniae* serotype 23  (strain BS 72) | N/A | Clinical isolate ([2](#_ENREF_2)) |
| *SP 0675* mutant | SP serotype 23 (BS 72) | *SP 0675* disrupted by tetracycline cassette |
| *SP 0320* mutant | SP serotype 23 (BS 72) | *SP 0320* disrupted by erythromycin cassette |
| *SP 0317* mutant | SP serotype 23 (BS 72) | *SP 0317* disrupted by tetracycline cassette |
| *SP 0318* mutant | SP serotype 23 (BS 72) | *SP 0318* disrupted by tetracycline cassette |
| *SP 0319* mutant | SP serotype 23 (BS 72) | *SP 0319* disrupted by tetracycline cassette |
| *SP 0320:0675* double mutant | SP serotype 23 (BS 72) | *SP 0320* disrupted with erythromycin cassette and *SP 0675* disrupted with tetracycline cassette |
| Complemented *SP 0320* mutant | *SP 0320* mutant | spectinomycin resistance cassette between s*pr 0112* and *spr 0113*, and *SP 0320* promoter with *SP 0320* ORF |
| Complemented *SP 0675* mutant | *SP 0675* mutant | spectinomycin resistance cassette between s*pr 0112* and *spr 0113,* and *SP 0675* promoter with *SP 0675* ORF |
| Complemented *SP 0317* mutant | *SP 0317* mutant | spectinomycin resistance cassette between s*pr 0112* and *spr 0113*, and *SP 0320* promoter with *SP 0317* ORF |

SP- *S. pneumoniae*. N/A – not applicable, ORF- open reading frame
