# Supplementary material for: Deletion of genes involved in the ketogluconate metabolism, Entner-Doudoroff pathway, and glucose dehydrogenase increase local and invasive virulence phenotypes in Streptococcus pneumoniae
Source: PLoS One. 2019 Jan 8;14(1):e0209688. doi: 10.1371/journal.pone.0209688 (PMC6324787; doi:10.1371/journal.pone.0209688)
Supplement: S4 Table — (DOCX) [file pone.0209688.s004.docx]

| ***S. pneumoniae* TIGR 4 or R6 Locus Number** | ***S. pneumoniae* serotype 23 (BS 72) Accession Number** |
| --- | --- |
| *SP 0317* (TIGR 4) | *ZP_01834468.1* |
| *SP 0318* (TIGR 4) | *ZP_01834469.1* |
| *SP 0319* (TIGR 4) | *ZP_01834470.1* |
| *SP 0320* (TIGR 4) | *ZP_01834471.1* |
| *SP 0675* (TIGR 4) | *ZP_01835181* |
| *Spr 0112* (R6) | *ZP_01834243.1* |
| *Spr 0113* (R6) | *ZP_01834246.1* |
