# Supplementary material for: Deletion of genes involved in the ketogluconate metabolism, Entner-Doudoroff pathway, and glucose dehydrogenase increase local and invasive virulence phenotypes in Streptococcus pneumoniae
Source: PLoS One. 2019 Jan 8;14(1):e0209688. doi: 10.1371/journal.pone.0209688 (PMC6324787; doi:10.1371/journal.pone.0209688)
Supplement: S5 Table — Table A. Primers used for mutation and confirmation (Restriction sites are in bold). Table B. Primers used for making complemented mutants and for confirmation (Restriction sites are in bold). Table C. Primers used for amplifying SP 0317, SP 0318, SP 0319, SP 0320 and SP 0675 to construct over expression plasmids and for confirmation (Restriction sites are in bold). Table D. Primers and probes used for DDPCR. (DOCX) [file pone.0209688.s005.docx]

Table A

| **Primer name** | **Sequence (5’ → 3’)** |
| --- | --- |
| SP 0317-Flnk1- F | GCTCCGTGAGGTGCGAGACAAA |
| **SP 0317-Flnk1-ApaI-R** | ATAT**GGGCCC**CTAACGGGTGGTTTATCTCAGAGCTA |
| **SP 0317-Flnk2-BamHI-F** | ATAT**GGATCC**CAAGCAATCGAGGCTTGTAGTCCTTCT |
| **SP 0317-Flnk2-R** | CGATCGAATCGGCCTCTACTATTT |
| **SP 0318-Flnk1- F** | TGTTTTAGAAGCAGAGGTGTACTATTCCAG |
| **SP 0318-Flnk1-ApaI-R** | ATAT**GGGCCC**GAATTACATGACCAAATCAGATACGAT |
| **SP 0318-Flnk2-BamHI-F** | ATAT**GGATCC**GCCAAAATAAGTCGAACTGGCAACATGATCG |
| **SP 0318-Flnk2-R** | CAATTTTGTCAATGGCCACATCTT |
| **SP 0319-Flnk1- F** | GGAATGATGGAGTAGTTGGTAAAGGG |
| **SP 0319-Flnk1-ApaI-R** | ATAT**GGGCCC**GGAACTAATGGATGACGAAAATATTACTGT |
| **SP 0319-Flnk2-BamHI-F** | ATAT**GGATCC**TTGGTAGCCTTTTTTATCTGTCGCTTC |
| **SP 0319-Flnk2-R** | GGAATTGGATTTGCTATTGCCA |
| **SP 0320-Flnk1- F** | GATACTGGTGGCATCTAATGGTGA |
| **SP 0320-Flnk1-BamHI-R** | TACC**GGATCC**GTCAATGGCCACATCCTATAT |
| **SP 0320-Flnk2-ApaI-F** | CTCC**GGGCCC**GACCAGATCTTGATTGATAT |
| **SP 0320-Flnk2-R** | CCATTTACGACTCCGCCCTG |
| **SP 0675-Flnk1-F** | CCGCAACGTTTCAAGTCTCGCCCTG |
| **SP 0675-Flnk1-ApaI-R** | CTCC**GGGCCCC**ATTTCTTGAGCTAGACCACC |
| **SP 0675-Flnk2-BamHI-F** | TACC**GGATCC**GAACCAGATGCAGTGGCTAAA |
| **SP 0675-Flnk2-R** | CGCAATCCCAAGGGAATCAC |
| **PcErm-ApaI-F** | CC**GGGCCC**AAAATTTGTTTGAT |
| **PcErm-BamHI-R** | TACC**GGATCC**AGTCGGCAGCGACTCATAGAAT |
| **PcTet-ApaI-F1** | CC**GGGCCC**AAAATTTGTTTGAT |
| **PcTet-BamHI-R1** | TACC**GGATCC**TCCCAAAGTTGATCCCTTAACGA |
| **PcTet-ApaI-F2** | ATCC**GGGCCC**AAAATTTGTTTGAT |
| **PcTet-BamHI-R2** | ATAT**GGATCC**TCCCAAAGTTGATCCCTTAACG |
| **SP 0317-Mutn-Confirm-F** | ATCGAGTGGATGATTTCTTCATGAG |
| **SP 0317-Mutn-Confirm-R** | CACCAGCCCCTTGAATAAAACG |
| **SP 0318-Mutn-Confirm-F** | CAAGTGCAGTCGTGATCTCTGTGA |
| **SP 0318-Mutn-Confirm-R** | GCCCCTTGAATAAAACGAGCCA |
| **SP 0319-Mutn-Confirm-F** | AGAGAATCCATATCTAGCATGTTTGGC |
| **SP 0319-Mutn-Confirm-R** | CCCCTTGAATAAAACGAGCCATAAT |
| **SP 0320-Mutn-Confirm-F** | GCAAGCATAGCCCCTACACC |
| **SP 0320-Mutn-Confirm-R** | CTGCTGACATTCCTTCCACA |
| **SP 0675-Mutn-Confirm-F** | GTCCACTTATGGCAAGGGTG |
| **SP 0675-Mutn-Confirm-R** | GGCGAACGCTTTATAGACTATC |

Table B

| **Primer name** | **Sequence (5’ → 3’)** |
| --- | --- |
| **SP 0317-F** | ATGACCAAATCAGATACGATTA |
| **SP 0317-R** | CGTTATCTGAGTGTAATATACTGT |
| **Prom-SphI-F** | ATAT**GCATGC**TCTTTTCTCCTTTCTTTTTAGG |
| **Prom-NcoI-R** | ATAT**CCATGG**TGATACTTCCTTTCAAATTTGT |
| **Spr 0112 flnk1-F** | GCATTCCTGTATCATAAAATTACAGAGTATGTC |
| **Spr 0112 flnk1-NcoI-R** | ATAT**CCATGG**TTGATAAAGCAAAGCTTTAAACCCTTCC |
| **Spr 0113 flnk 2-NdeI-F** | AGCT**CATATG**TATTATAGCTCTTGGTATGATATTGGC |
| **Spr 0113 flnk2-R1** | GTATTCTGAGTATCGTGCAGATGAT |
| **PcSpec*-*NcoI-F** | CCAT**CCATGG**CCGTTTGATTTTTAATGGTAATG |
| **PcSpec-SphI-R** | ATAT**GCATGC**GACGCGGAATGGATCCAATTTT |
| **SP 0317-Comple-Confirm-F** | ACCGTACATTCCAGGCTGTATT |
| **SP 0317-Comple-Confirm-R** | GAATGACTGTTGCAATGAAGTA |
| **SP Prom-0320-F** | TCTTTTCTCCTTTCTTTTTAGGTTTACCGGTTTACT |
| **SP Prom-0320-R** | TACTCAGGTTGTTTTCCGATGT |
| **Spr 0113 flnk2-SpeI-F** | CCAT**ACTAGT**TATTATAGCTCTTGGTATGATATTGGC |
| **Spr 0113-flnk2-R2** | GTATTCTGAGTATCGTGCAGATGAT |
| **SP 0320-Comple-Confirm-F** | ATAGCTATGAAACATTTTTTTGCAGGAATTGGTGAA |
| **SP 0320-Comple-Confirm-R** | ATTCTTGTTTGGGAGGATGATT |
| **SP 0675-F** | ATGCCTACTATTCTCATTACCG |
| **SP 0675-R** | TTACTTATAATTAAAAGTTTCC |
| **Ami-SphI-F** | ATAT**GCATGC**CGATAAGCTTGATGAAAATTTG |
| **Ami-SacII-R** | ATAT**CCGCGG**TATGCCTCCTAAATTTTTATCT |
| **Ami-0675-HindII-F** | CGAT**AAGCTT**GGGCGAATTGGGCCCGACGTCGCATGCTCGATAAGC |
| **Ami-0675-PstI-R** | ATAT**CTGCAG**TATGGTCGACCTGCAGGCGGCC |
| **Spr 0112-flnk1-SphI-R** | ATAT**GCATGC**TTGATAAAGCAAAGCTTTAAACCCTTCC |
| **Spr 0113 flnk2-PstI-F** | ATAT**CTGCAG**TATTATAGCTCTTGGTATGATATTGGC |
| **PcSpec-SphI-F** | ATAT**GCATGC**CCGTTTGATTTTTAATGGTAATG |
| **PcSpec-HindIII-R** | ATAT**AAGCTT**GACGCGGAATGGATCCAATTTT |
| **SP 0675-Comple-Confirm-F** | AGGTGTAGACTTAAATTTTGGA |
| **SP 0675-Comple-Confirm-R** | TCAATGGAAAATAAAATATTGC |

Table C

| **Primer name** | **Sequence (5’ → 3’)** |
| --- | --- |
| **SP 0317-Pml I-F** | ATAT**CACGTG**ATGACCAAATCAGATACGATTATTGAAC |
| **SP 0317-Avr II-R** | ATAT**CCTAGG**TTATCTGAGTGTAATATACTGT |
| **SP 0318-Pml I-F** | ATAT**CACGTG**ATGACGAAAATATTACTGTTTGGCGAAC |
| **SP 0318-Avr II-R** | ATAT**CCTAGG**CTAACGAATGATATCTTGTGCA |
| **SP 0319-Pml I-F** | ATAT**CACGTG**ATGAAAATCGCATTAATCAATGAAAATAGTCAAGC |
| **SP 0319-Avr II-R** | ATAT**CCTAGG**TTACTTGGCTAATACTTCTTTC |
| **SP 0320-Pml I-F** | ATAT**CACGTG**ATGACAAATACATCATTCTCAATTG |
| **SP 0320-Avr II-R** | ATAT**CCTAGG**TTACTCAGGTTGTTTTCCGATG |
| **SP 0675-Pml I-F** | ATAT**CACGTG**ATGCCTACTATTCTCATTACCG |
| **SP 0675-Avr II-R** | ATAT**CCTAGG**TTACTTATAATTAAAAGTTTCC |
| **pET-SP 0317- Confirm-F** | CCGCGAAATTAATACGACTCACTAT |
| **pET-SP 0317- Confirm-R** | CCTGATACAAGTCTACAAGTTCCTTGATG |
| **pET-SP 0318- Confirm-F** | AATTAATACGACTCACTATAGGGGAATTG |
| **pET-SP 0318- Confirm-R** | TGAGAAAACGATCTCCAATCTCG |
| **pET-SP 0319- Confirm-F** | GCGGATAACAATTCCCCTCTAGA |
| **pET-SP 0319- Confirm-R** | CCACAGCCGGTAACAACAAAGT |
| **pET-SP 0320- Confirm-F** | ATTAATACGACTCACTATAGGGGAATTGTG |
| **pET-SP 0320- Confirm-R** | GATGCCAACTTCACGATAAGCTTC |
| **pET-SP 0675- Confirm-F** | AATTAATACGACTCACTATAGGGGAATTG |
| **pET-SP 0675- Confirm-R** | GGAGATAAAGATCAGTTACCAGAGCTTC |

Table D

| **Primer name** | **Sequence (5’ → 3’)** |
| --- | --- |
| **Hyl-F** | GTG GAC AGG AAG CAA AGC TC |
| **Hyl-R** | CTT TGG CAA TCC CGA TTT TA |
| **RegR-F** | GAA ATC GAT CCC GAT GAA AA |
| **RegR-R** | AGC CTG TTG TCC TTC CTC AA |
| **GyrB-F** | TTT CTC CGA TTT CCT CAT GG |
| **GyrB-R** | CCA CCA GCT GAG TCT CCT TC |
| **Hyl-probe** | /5HEX/TGC CTC AAC TCG AGT CAT TG/3BHQ_1/ |
| **RegR-probe** | /5HEX/AAC TGT TGG GCC CTA CCT CT/3BHQ_1/ |
| **GyrB-probe** | /56-FAM/TCC ACA GAT TGC CAA ACG TA/3BHQ_1/ |
