# Supplementary material for: Deletion of genes involved in the ketogluconate metabolism, Entner-Doudoroff pathway, and glucose dehydrogenase increase local and invasive virulence phenotypes in Streptococcus pneumoniae
Source: PLoS One. 2019 Jan 8;14(1):e0209688. doi: 10.1371/journal.pone.0209688 (PMC6324787; doi:10.1371/journal.pone.0209688)
Supplement: S6 Table — (DOCX) [file pone.0209688.s006.docx]

| **Plasmid/ DNA** | **Description** | **Source** |
| --- | --- | --- |
| **PcErm** | Template for amplifying Erm^R^ cassette | UIC |
| **PcTet** | Template for amplifying Tet^R^ cassette | UIC |
| **pR412** | Template for amplifying Spec^R^ cassette | UIC |
| **pEVP3** | Plasmid template used for amplifying the Ami promoter | UIC |
| **pGEMT:0317** | 0317 cloned into the EcoRV site of pGEMT easy | This work |
| **pGEMT:Prom:0317** | Promoter cloned into SphI and NcoI sites upstream of 0317 on pGEMT:0317 | This work |
| **pGEMT:Prom:0320** | 0320 cloned into the EcoRV site of pGEMT easy along with self promoter | This work |
| **pGEMT:0675** | 0675 cloned into the EcoRV site of pGEMT easy | This work |
| **pGEMT:Ami:0675** | Ami promoter cloned into SphI and SacII sites upstream of 0675 on pGEMT:0675 | This work |
